# Pharmacologic ATF6 activation confers global protection in widespread disease models by reprograming cellular proteostasis

**DOI:** 10.1038/s41467-018-08129-2

**Published:** 2019-01-14

**Authors:** Erik A. Blackwood, Khalid Azizi, Donna J. Thuerauf, Ryan J. Paxman, Lars Plate, Jeffery W. Kelly, R. Luke Wiseman, Christopher C. Glembotski

**Affiliations:** 1 San Diego State University Heart Institute and the Department of Biology, San Diego State University, San Diego, CA 92182 USA; 2 Department of Chemistry, The Scripps Research Institute, La Jolla, CA 92037 USA; 3 Department of Molecular Medicine, The Scripps Research Institute, La Jolla, CA 92037 USA

## Abstract

Pharmacologic activation of stress-responsive signaling pathways provides a promising approach for ameliorating imbalances in proteostasis associated with diverse diseases. However, this approach has not been employed in vivo. Here we show, using a mouse model of myocardial ischemia/reperfusion, that selective pharmacologic activation of the ATF6 arm of the unfolded protein response (UPR) during reperfusion, a typical clinical intervention point after myocardial infarction, transcriptionally reprograms proteostasis, ameliorates damage and preserves heart function. These effects were lost upon cardiac myocyte-specific *Atf6* deletion in the heart, demonstrating the critical role played by ATF6 in mediating pharmacologically activated proteostasis-based protection of the heart. Pharmacological activation of ATF6 is also protective in renal and cerebral ischemia/reperfusion models, demonstrating its widespread utility. Thus, pharmacologic activation of ATF6 represents a proteostasis-based therapeutic strategy for ameliorating ischemia/reperfusion damage, underscoring its unique translational potential for treating a wide range of pathologies caused by imbalanced proteostasis.

## Abstract (web summary)

Imbalanced proteostasis is associated with diverse diseases, including ischemia/reperfusion injury in the heart. Here the authors show that the ATF6 arm of the unfolded protein response can be pharmacologically activated with a small molecule in vivo, providing protection from ischemia/reperfusion injury in the heart, the brain, and the kidney.

## Introduction

Protein homeostasis, or proteostasis is maintained by pathways that coordinate protein synthesis and folding with the degradation of misfolded, potentially toxic proteins^1,2^. Endoplasmic reticulum (ER) proteostasis is particularly important, since nearly one-third of all proteins are made and folded in the ER, then transported to their final destinations as integral membrane or soluble secreted proteins^3^. Imbalances in proteostasis cause or exacerbate numerous pathologies, spawning interest in the exogenous manipulation of proteostasis as a therapeutic approach for such diseases^4^. ER proteostasis is regulated by the unfolded protein response (UPR), a stress-responsive signaling pathway comprising three sensors/effectors of ER protein misfolding; PERK (protein kinase R [PKR]-like ER kinase), IRE1 (inositol-requiring enzyme 1), and ATF6 (activating transcription factor 6)^5^. Considerable evidence supports ATF6, a transcriptional regulator of ER proteostasis, as a viable therapeutic target for exogenous manipulation of proteostasis^6–11^; however, such an approach has not been examined in vivo. Accordingly, here, we determined whether treatment with a pharmacological activator of ATF6 would reprogram proteostasis and mitigate pathology in a mouse model of ischemic diseases, such as those that affect the heart.

Ischemic heart disease is the leading cause of human deaths worldwide^12^. These deaths are mainly due to acute myocardial infarction (AMI), where thrombotic coronary artery occlusion causes rapid, irreparable ischemic injury to the heart, increasing susceptibility to progressive cardiac degeneration and eventual heart failure^13–15^. The treatment of choice for AMI is primary percutaneous coronary intervention, or coronary angioplasty^16^, which results in reperfusion. While reperfusion limits ischemic injury, the reperfusion itself injures the heart, in part by increasing reactive oxygen species (ROS). ROS contribute to AMI injury, also known as ischemia/reperfusion (I/R) injury, mainly by damaging proteins, which impairs proteostasis^17,18^. In fact, reperfusion accounts for up to 50% of the final damage from AMI^19^; however, there is no clinically available intervention that mitigates reperfusion injury at the time of coronary angioplasty, underscoring the importance of developing therapies that reduce ROS during reperfusion^19^. Using a mouse model of global *Atf6* deletion, we recently showed that, in the heart, ATF6 is responsible for the expression of a broad spectrum of genes not traditionally identified as regulated by ATF6, including many antioxidant genes that could improve proteostasis during I/R^10^. While this genetic approach identified the potential importance of ATF6 as a novel therapeutic target for pharmacological intervention in I/R injury models, there have been no reports addressing whether a single arm of the UPR can be pharmacologically activated and shown to be beneficial in any animal model of pathology.

We recently identified a compound that we call **147** in a high-throughput cell-based reporter screen, where it was shown to selectively induce only the ATF6 arm of the UPR^20^. Here, we examined the effects of pharmacological activation of ATF6 with **147** in a mouse model of AMI. We found that intravenous administration of **147** concurrently with AMI robustly and selectively activated ATF6 and downstream genes of the ATF6 gene program and protected the heart from I/R damage; however, this protection was lost upon the genetic deletion of *Atf6*. Moreover, **147** had no deleterious effects in the absence of pathology, or in other tissues that were unaffected by I/R, an indicator of its safety. Remarkably, we found that by activating ATF6, **147** protected other tissues, including the brain, kidney, and liver, when they were subjected to maneuvers that induced I/R damage and impaired proteostasis. Thus, **147** selectively activates the ATF6 arm of the UPR in vivo, exhibiting significant potential as a therapeutic approach for treating I/R damage in a wide range of tissues.

## Results

### ATF6 in cardiac myocytes protects the heart from I/R injury

**Fig. 1:**
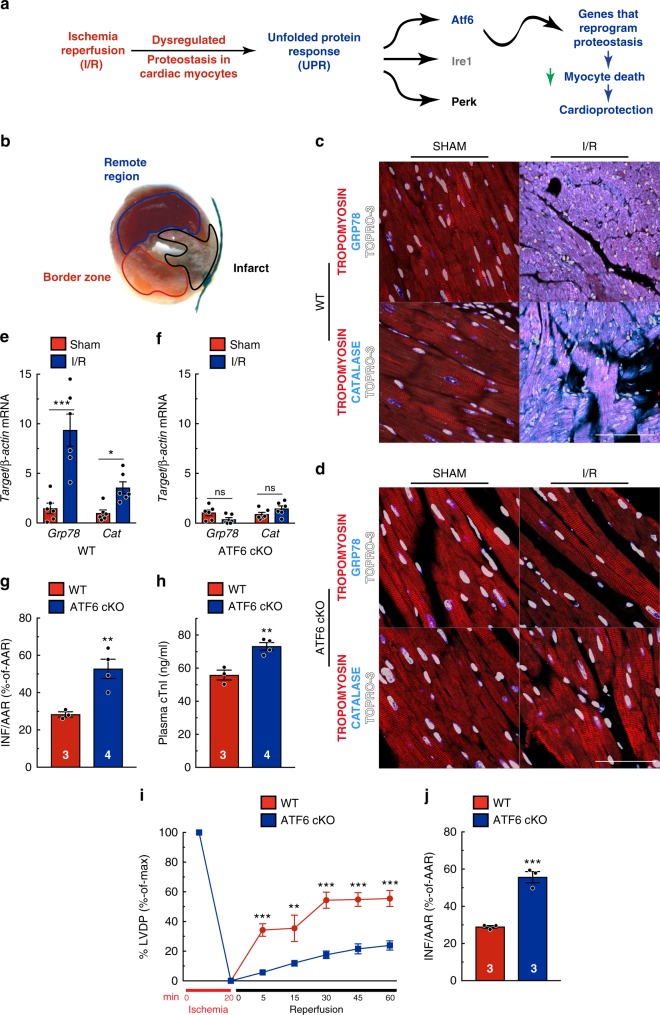
ATF6 in cardiac myocytes protects the heart from I/R injury. **a** Diagram of the hypothetical activation of the unfolded protein response (UPR) by ischemia/reperfusion (I/R) in the heart. **b** Post AMI cross-section of the left ventricle of a mouse heart after I/R and TTC staining to identify the infarct region (black), border zone (red) CAT (cyan), tropomyosin (red), and nuclei (TOPRO-3, white) in the border zone of wild-type (WT) (**c**) or ATF6 cKO (**d**) hearts subjected to either sham or I/R surgery with 24 hours of reperfusion. Tissue sections are representative images from one mouse per condition. Scale bar represents 50 µm. **e**, **f** Quantitative real-time PCR (qPCR) for *Grp78* or *Cat* in sham or border zone of post-I/R hearts in WT (*n* = 6) (**e**), ATF6 cKO (*n* = 6) (**f**). **g**, **h** Infarct sizes (**h**) and plasma cardiac troponin I (cTnI) (**i**) in WT (*n* = 3) and ATF6 cKO (*n* = 4) mice post I/R. **i**, **j** Left ventricular developed pressure (LVDP) (**i**) and relative infarct sizes (**j**) post ex vivo I/R (*n* = 3). Data are represented as mean ± s.e.m. Two-group comparisons were performed using Student’s two-tailed *t* test, and all multiple group comparisons were performed using a one-way ANOVA with a Newman–Keuls post hoc analysis. *P *≤* 0.05, **P *≤* 0.01, and ***P *≤* 0.001; ns not significant

Given their roles in contraction, the viability of cardiac myocytes is crucial for heart function, and cardiac myocyte death during I/R leads to impairment of this function^17^. Accordingly, we examined the effects of I/R on proteostasis in isolated cardiac myocytes and in the mouse heart, positing that I/R disrupts proteostasis, leading to activation of all three arms of the UPR, and that the ATF6 arm induces genes that adaptively reprogram proteostasis, decrease myocyte death, and provide cardioprotection from I/R damage (Fig. 1a). Consistent with this hypothesis was our finding that I/R activated ATF6, as well as the IRE1 and PERK arms of the UPR in cultured cardiac myocytes, albeit to a lesser extent than the chemical activator of the UPR, tunicamycin (TM) (Supplementary Figure 1a–d). As a measure of ATF6 activation, we examined the expression of two known ATF6 target genes, glucose-regulated protein 78 kDa (*Grp78*), a well-studied ER heat-shock protein 70 chaperone, also known as BiP^21^, which participates in ER protein folding, and catalase (*Cat*), a prominent member of a novel antioxidant gene program recently shown to be induced by ATF6^10^. In accordance with the increased activity of ATF6 in response to I/R, both *Grp78* and *Cat* were induced in cultured cardiac myocytes (Supplementary Figure 1a, e, f, g).

To examine the effects of deleting *Atf6* specifically from cardiac myocytes, in vivo, we made an *Atf6* conditional knockout (ATF6 cKO) mouse in which *Atf6* was selectively deleted in cardiac myocytes of ATF6^fl/fl^ mice using adeno-associated virus serotype 9 (AAV9)-cTnT-CRE (Supplementary Figure 2a, b). ATF6 cKO and wild-type (WT) mice, the latter of which retain *Atf6*, were subjected to 30 min of surgical coronary artery ligation, followed by 24 hours of reperfusion (I/R), which mimics the reperfusion injury in AMI patients that occurs acutely, a time during which the extent of reperfusion injury is progressive^22^. In this model, I/R causes cardiac myocyte death and irreparable damage in the infarct zone (Fig. 1b, black), where blood flow has been completely occluded. However, cardiac myocytes adjacent to the infarct, in the border zone (Fig. 1b, red), are exposed to sub-lethal I/R and mount protective stress responses, such as the UPR, while the remote region (Fig. 1b, blue) is relatively unaffected^13,23^. Thus, protective stress responses in border zone myocytes conserve their viability, thereby reducing the size of the infarct. WT mice exhibited a robust activation of ATF6 in response to I/R, as evidenced by induction of the ATF6 target genes, *Grp78* and *Cat*, in the border zone of hearts subjected to acute I/R (Fig. 1c, e); however, this induction was lost in ATF6 cKO mice (Fig. 1d, f). In contrast, the IRE1 target gene, *Erdj4*, and PERK target gene, *Atf4*, were similarly induced by I/R in WT and ATF6 cKO mouse hearts (Supplementary Figure 2c, d). However, compared to WT, ATF6 cKO mice had increased infarct sizes and plasma cardiac troponin I (cTnI) (Fig. 1g, h), canonical indicators of cardiac injury, and exhibited increased lipid peroxidation (Supplementary Figure 2e), a measure of ROS-mediated damage. Cardiac hemodynamics were also assessed in an ex vivo isolated perfused heart model that enables the precise measurement of the strength of cardiac pump function, that is, left ventricular developed pressure (LVDP), with each contraction in response to I/R injury^10^. ATF6 cKO mouse hearts exhibited significantly lower recovery of LVDP and larger infarcts than WT hearts (Fig. 1i, j). Collectively, these results show that ATF6 in cardiac myocytes protects the heart from I/R injury. Thus, while all three arms of the UPR were activated in the ischemic mouse heart, cardiac specific deletion of *Atf6* significantly increased heart damage in response to I/R, demonstrating the importance of the ATF6 arm of the UPR in mitigating I/R injury in the heart.

**Fig. 2:**
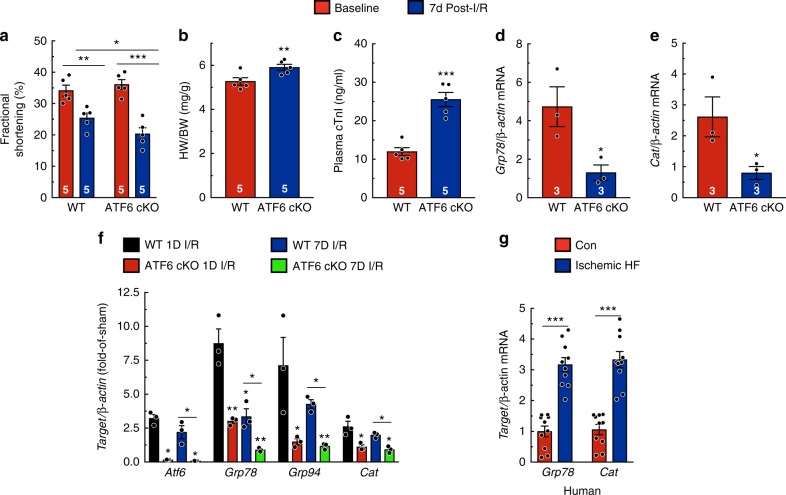
Endogenous ATF6 is cardioprotective in a model of a chronic AMI. **a**–**e** Parameters from mice 7 days post I/R. **a** Fractional shortening. Detailed analyses of echocardiography parameters are in Extended Data Table 1 (*n* = 5). **b** Ratio of heart weight to body weight. **c** Plasma cTnI. **d**, **e** qPCR for *Grp78* (**d**) or *Cat* (**e**) in border zone of mice (*n* = 3). **f** qPCR for *Atf6* and ATF6 target genes *Grp78*, *Grp94*, and *Cat* in WT (*n* = 3) and ATF6 cKO (*n* = 3) mice either 1 day or 7 days after I/R. **g** Quantitative real-time PCR (qPCR) for *Grp78* or *Cat* in ventricular explants from control (*n* = 10) or ischemic heart failure (*n* = 10) patients. Data are represented as mean ± s.e.m. Two-group comparisons were performed using Student’s two-tailed *t* test, and all multiple group comparisons were performed using a one-way ANOVA with a Newman–Keuls post hoc analysis. *P *≤* 0.05, **P *≤* 0.01, and ***P *≤* 0.001

In the days following AMI, the infarct continues to expand and remodels to become a fibrotic scar, so the detrimental effects of I/R on cardiac function and performance are often more pronounced a week after infarction^13^. Therefore, to examine the effect of *Atf6* deletion on cardiac function and performance, mice were analyzed 7 days after AMI. ATF6 cKO mice exhibited significantly reduced fractional shortening compared to WT, despite being aphenotypic at baseline (Fig. 2a; Supplementary Table 1). ATF6 cKO mice also exhibited exaggerated pathological cardiac hypertrophy and increased plasma cTnI (Fig. 2b, c). Notably, the levels of *Grp78* and *Cat* were lower in ATF6 cKO than WT mice at 7 days (Fig. 2d, e). When gene expression was examined at 1 and 7 days after AMI, the induction of *Atf6* and its target genes remained increased through 7 days in WT and less so in ATF6 cKO mouse hearts, although the level of induction was lower at 7 days compared to 1 day post AMI (Fig. 2f), indicating that the adaptive effects of ATF6-induced genes are likely exerted for at least the first week following AMI. *Grp78* and *Cat* were also increased in hearts from patients with ischemic heart disease (Fig. 2g), supporting the relevance of the ATF6 adaptive arm of the UPR in human pathology and validating the phenotypes observed in this mouse model of AMI.

Interestingly, I/R activated ATF6 less than TM, the latter of which is a strong, chemical inducer of ER protein misfolding and activator of the UPR (Supplementary Figure 1a). Importantly, this result suggests that during I/R there is a reserve of inactive ATF6 that has the potential to be activated. Accordingly, we hypothesized that selective pharmacologic activation of ATF6 could supplement the modest ATF6 activation achieved upon I/R, and that this supplemental ATF6 activation might enhance cardioprotection.

### 147 activates ATF6 and its target genes in cardiac myocytes

**Fig. 3:**
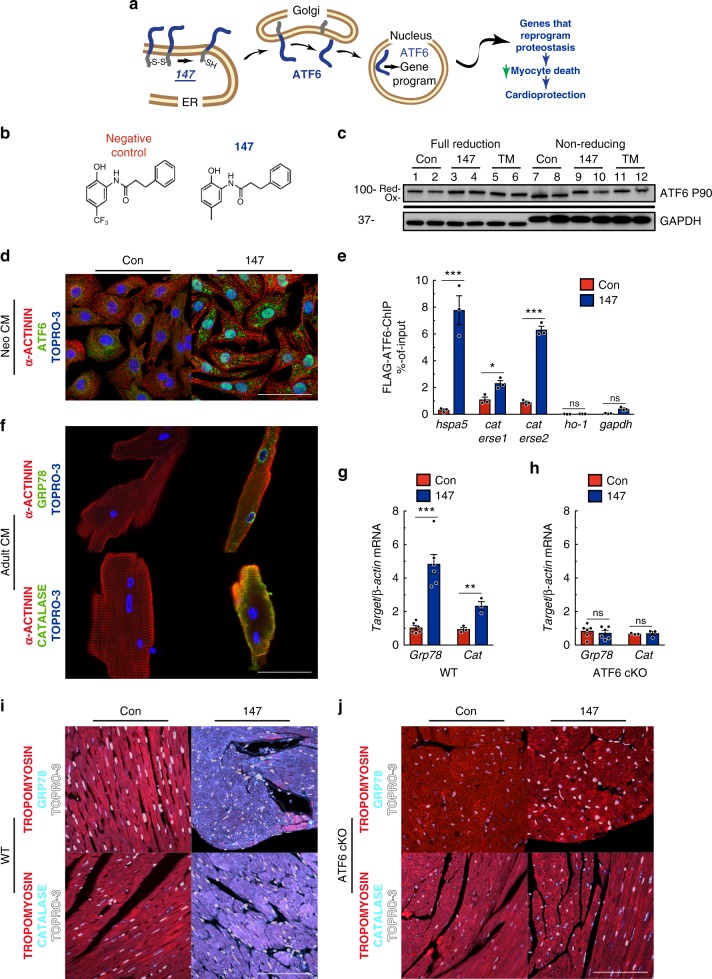
**147** selectively activates ATF6 in the heart. **a** Diagram of the hypothetical mechanism of ATF6 activation by **147**. **b** Chemical structure of synthetic control compound and compound **147**. **c** Immunoblot of ATF6 and GAPDH in NRVM 24 hours after treatment with compound **147** or TM in fully reducing condition (lanes 1–6) or non-reducing conditions (lanes 7–12). Shift exhibited in Atf6 in TM-treated cells in full-reducing conditions is typical of de-glycosylated ATF6. Full uncut version of this gel can be found in Supplementary Figure 7**. d** Immunocytofluorescence (ICF) of ATF6 (green), α-actinin (red), and nuclei (TOPRO-3) in NRVM 24 hours after treatment with compound **147**. Scale bar represents 50 µm. **e** Chromatin immunoprecipitation (ChIP-qPCR) of known ATF6 target promoter binding elements (ERSE) for Grp78 (*hspa5*), *cat*, and negative control targets Heme oxygenase 1 (*ho-1*) and *gapdh* NRVM infected with AdV encoding Flag-ATF6 (1–670) 24 hours after treatment with compound **147** (*n* = 3). **f** ICF of GRP78 and CAT (green), α-actinin (red), and nuclei (TOPRO-3) in AMVM 24 h after treatment with compound **147**. **g**, **h** qPCR for *Grp78* (*n* = 6) or *Cat* (*n* = 3) in LV of WT (**g**) or ATF6 cKO (**h**) hearts 24 hours post treatment with control or **147**. **i**, **j** IHC staining of GRP78 or CAT (cyan), tropomyosin (red), and nuclei (TOPRO-3, white) in left ventricle (LV) of WT (**i**) or ATF6 cKO (**j**) hearts 24 hours post treatment with control or **147**. Tissue sections are representative images from one mouse per condition. Scale bar represents 50 µm. Data are represented as mean ± s.e.m. Two-group comparisons were performed using Student’s two-tailed *t* test, and all multiple group comparisons were performed using a one-way ANOVA with a Newman–Keuls post hoc analysis. *P *≤* 0.05, **P *≤* 0.01, and ***P *≤* 0.001

The compound **147** was previously shown to specifically activate ATF6 in HEK293 cells through a mechanism involving translocation of ATF6 from the ER to the Golgi, where it is cleaved by S1 and S2 proteases to release the active ATF6 transcription factor^20^ (Fig. 3a). The translocation of ATF6 out of the ER during protein misfolding is known to require a reduction of the inter- and intramolecular disulfide bonds in ATF6; however, neither the effects of **147** on ATF6 nor its mechanism of action have been studied in cardiac myocytes. Here, in cultured neonatal rat ventricular myocytes (NRVM), a control compound that closely resembles **147** (Fig. 3b), but does not activate ATF6, did not affect the disulfide bond status of ATF6, while **147** reduced intramolecular disulfide bonds in ATF6 (Fig. 3c, lanes 7–10). Moreover, while the control compound did not activate any of the UPR pathways, **147** activated ATF6, but not PERK or IRE1 (Supplementary Figure 3a-d). Thus, in cardiac myocytes, **147** induced the reduction of disulfide bonds in ATF6, which is associated with ATF6 translocation to the Golgi. Coordinate with the generation of the active, nuclear form of ATF6 in the Golgi was our finding that **147** increased the nuclear translocation of ATF6 in cardiac myocytes (Fig. 3d) and increased the specific cleavage and activation of ATF6 (Supplementary Figure 3a, b, g). Mechanistically, **147** increased the association of ATF6 with known ATF6 binding sites in the *Grp78* and *Cat* promoters (Fig. 3e), and **147** increased GRP78 and CAT (Supplementary Figure 3a, e, f). Intravenous administration of **147** activated ATF6 and increased *Grp78* and *Cat* expression in WT mouse hearts; however, this effect was completely absent in ATF6 cKO mice (Fig. 3g–j; Supplementary Figure 3h). As a testament to the ability of **147** to activate only the ATF6 arm of the UPR was our finding that **147** had no effect on the expression levels of the IRE1 or PERK targets, *Erdj4* or *Atf4* in either WT or ATF6 cKO mouse hearts (Supplementary Figure 3i, j). Thus, **147** selectively activates the ATF6 arm of the UPR in the heart, in vivo, as it does in cultured cardiac myocytes.

### 147 improves ER proteostasis and decreases oxidative stress

**Fig. 4:**
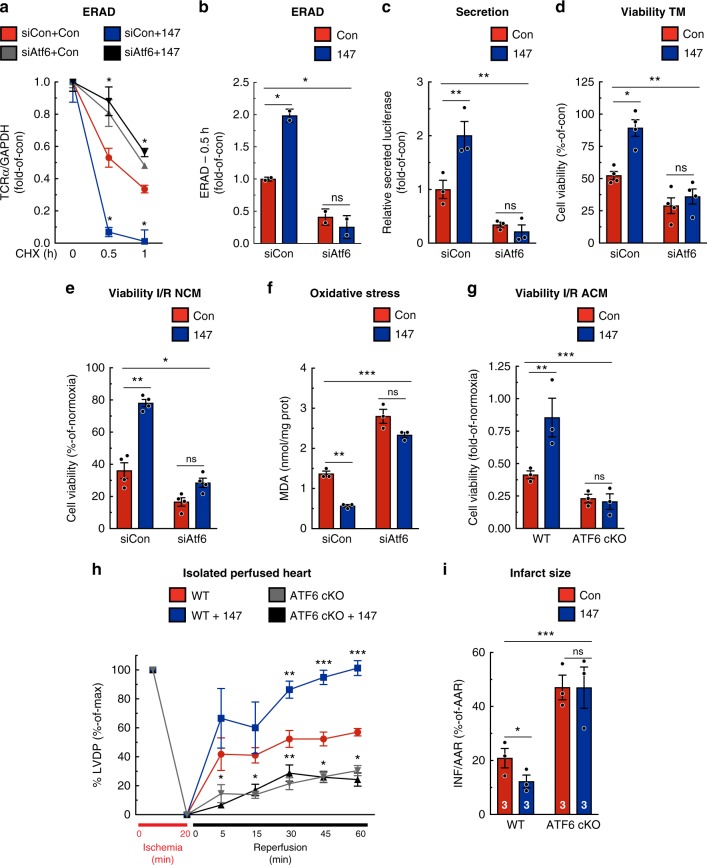
**147** improves proteostasis and decreases oxidative stress in an ATF6-dependent manner. **a**, **b** NRVM were infected with AdV-HA-T cell antigen receptor α-chain (TCRα; an ER-transmembrane protein that is chronically misfolded and degraded by ERAD), treated with siCon or siAtf6 and either control or **147** for 24 h prior to cyclohexamide for 0, 0.5, or 1 h. Densitometry of the HA-TCRα immunoblots at the respective times (**a**) and ERAD at the 0.5-h time point (**b**) are shown (*n* = 2). **c** Secretory proteostasis assayed in NRVMs when transfected with *Gaussia* luciferase and treated with siCon or siAtf6, and either control or **147** for 24 hours. Medium was collected and luciferase activity was measured (*n* = 3). **d** NRVMs were transfected with siCon or siAtf6, and then treated with or without TM, control or **147** for 24  hours, after which viability was determined (*n* = 4). **e**, **f** NRVMs were transfected with siCon or siAtf6, treated with or without control or **147** for 24 hours, and then I/R, after which viability (**e**) and malondialdehyde (MDA) (**f**) were measured. **g** Viability of I/R-treated cultured adult cardiomyocytes isolated from WT (*n* = 3) or ATF6 cKO (*n* = 3) mice 24 hours post treatment with control or **147**. **h**, **i** LVDP (**h**) and relative infarct sizes (**i**) of WT or ATF6 cKO mice treated 24 hours with control or **147** and then ex vivo I/R. Data are represented as mean ± s.e.m. Two-group comparisons were performed using Student’s two-tailed *t* test, and all multiple group comparisons were performed using a one-way ANOVA with a Newman–Keuls post hoc analysis. **P *≤* 0.01 and ***P *≤* 0.001; ns not significant

Mechanistically, we examined whether **147** could replicate the breadth of adaptive effects of ATF6 on ER proteostasis, such as increasing ER-associated protein degradation (ERAD), which removes potentially toxic terminally misfolded proteins, increasing folding and subsequent secretion of proteins made in the ER, and enhancing protection against ER protein misfolding. **147** increased ERAD, as measured by the rate of degradation of ectopically expressed T cell antigen receptor α-chain (TCRα)^24^ (Fig. 4a, b), increased the folding and secretion of protein from the ER pathway (Fig. 4c), and protected cells from death in response to ER protein misfolding induced by TM (Fig. 4d); importantly, all of these effects were lost upon knockdown of *Atf6*. Next, we explored whether **147** could replicate the adaptive effects of ATF6 against oxidative stress, in vitro. **147** significantly improved the survival of cardiac myocytes subjected to I/R (Fig. 4e) and decreased ROS-mediated damage (Fig. 4f). Importantly, these effects of **147** were lost upon siRNA-mediated knockdown of *Atf6*. Thus, **147** replicated a broad spectrum of the adaptive effects of ATF6 on proteostasis and oxidative stress. Moreover, all of these effects required endogenous ATF6, demonstrating the ATF6-dependent mechanism of action of **147**.

### 147 in vivo protects cardiac myocytes and hearts in vitro

To determine whether **147** can protect cardiac myocytes in vivo, mice were treated for 24 hours with either the negative control compound or **147**, after which cardiac myocytes were isolated and subjected to I/R in culture. Compared to the negative control, myocytes from **147**-treated WT mice exhibited increased viability when subjected to I/R in vitro (Fig. 4g, left); however, this benefit was absent in myocytes from ATF6 cKO mice (Fig. 4g, right). Thus, when administered in vivo, **147** protected cardiac myocytes from I/R damage in culture, and this protection was mediated through endogenous ATF6. To determine whether the protection seen in isolated cardiac myocytes had any effect in the intact heart, hearts from WT and ATF6 cKO mice that had been treated for 24 hours with **147** were examined in the ex vivo I/R model. Compared to control, hearts from **147-**treated WT mice had greater LVDP recovery and smaller infarct sizes (Fig. 4h, blue vs. red; 4i, left). Notably, **147** exhibited neither of these beneficial effects in hearts from ATF6 cKO mice (Fig. 4h, gray and black; 4i, right). Thus, when administered to mice, **147** protected cardiac myocytes, and decreased I/R injury of the heart while preserving cardiac function. Furthermore, all of these beneficial effects of **147** were dependent upon endogenous ATF6 in cardiac myocytes.

### 147 transiently activates ATF6 in the heart

**Fig. 5:**
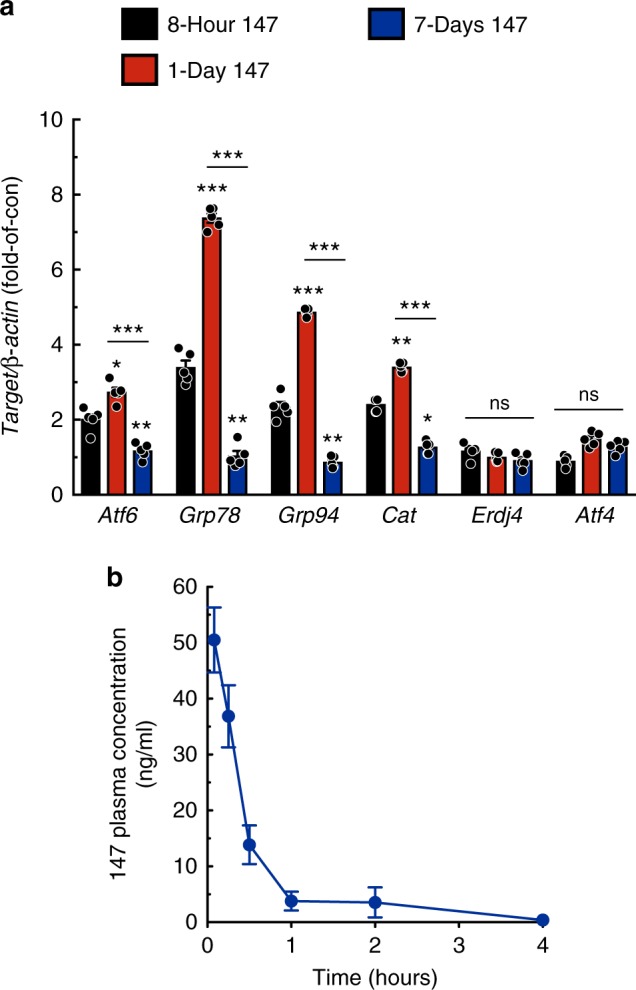
**147** acts transiently in vivo. **a** qPCR for *Atf6* and ATF6 target genes *Grp78*, *Grp94*, and *Cat* in WT (*n* = 5) mice either 8 hours, 1 day, or 7 days after a single bolus venous injection of **147** (2 mg/kg). **b** **147** plasma concentration as a function of time in mice receiving 2 mg/kg via venous injection. Blood was collected at baseline and 5min, 15min, 30min, 1h, 2h, and 4h post injection (*n* = 4 mice per timepoint). Data are represented as mean ± s.e.m. Two-group comparisons were performed using Student’s two-tailed *t* test, and all multiple group comparisons were performed using a one-way ANOVA with a Newman–Keuls post hoc analysis. *P *≤* 0.05, **P *≤* 0.01, and ***P *≤* 0.001; ns not significant

**Fig. 6:**
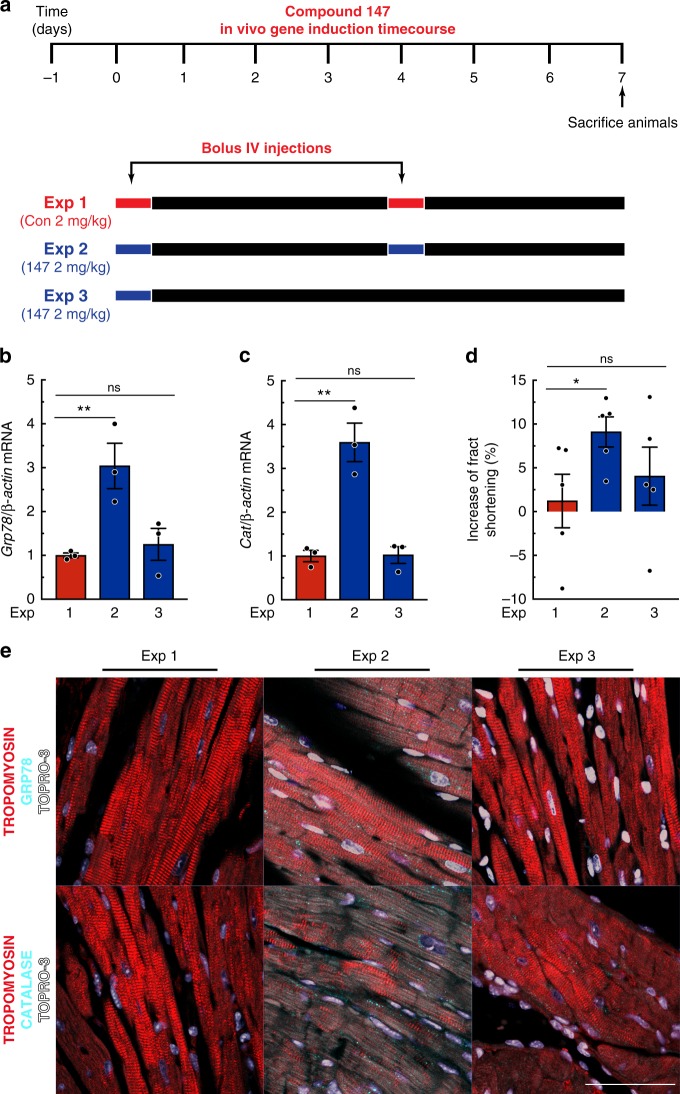
**147** gene induction timecourse in vivo. **a** Experimental design testing the effects of **147** in WT untreated mice. Red bars depict the bolus administration of the control compound, while blue bars depict the bolus administration of **147**. **b**, **c** qPCR for *Grp78* (**b**) or *Cat* (**c**) in LV of mice from indicated experiments (*n* = 3). **d** Percent increase in fractional shortening. Detailed analyses of echocardiography parameters are in Extended Data Table 2 (*n* = 5). **e** IHC staining of GRP78 or CAT (cyan), tropomyosin (red), and nuclei (TOPRO-3, white) in LV of mice from respective experiments. Tissue sections are representative images from one mouse per condition. Scale bar represents 50 µm. Data are represented as mean ± s.e.m. Two-group comparisons were performed using Student’s two-tailed *t* test, and all multiple group comparisons were performed using a one-way ANOVA with a Newman–Keuls post hoc analysis. *P *≤* 0.05 and **P *≤* 0.01; ns not significant

To begin to understand the temporal dynamics of the function of **147** in mice, a time course of gene induction was performed. *Atf6* and its target genes were induced at the earliest time point examined, 8 hours, reaching a maximum 24 hours after administration and falling back to baseline values by 7 days after administration (Fig. 5a). Furthermore, pharmacokinetic studies revealed a relatively rapid clearance of **147** from plasma (Fig. 5b), supporting the further examination of multiple **147** dosing strategies. Accordingly, several dosing strategies spanning 7 days were used to examine the temporal dynamics of the effects of **147** on ATF6 target gene induction in the hearts of mice that were not subjected to I/R using several dosing protocols spanning 7 days (Fig. 6a). Mice were injected with the negative control compound or **147** either twice, at days 0 and 4 (Experiments 1 and 2, respectively), or **147** was injected only once, at day 0 (Experiment 3). Compared to Experiment 1, Experiment 2 but not 3 resulted in the increased expression of the ATF6-regulated genes *Grp78* and *Cat* (Fig. 6b, c, e), but not the IRE1-regulated *Erdj4* or the PERK-regulated *Atf4* (Supplementary Figure 4a, b). These results indicated that **147**-mediated induction of ATF6 target genes is transient, as gene expression was increased 3 days after administration, but returned back to baseline 7 days after administration.

Interestingly, Experiment 2 significantly enhanced cardiac performance (Fig. 5d; Experiment 1 vs. 2; Supplementary Table 2), which could be partly due to **147**-dependent increases in *Atp2a2* expression (Supplementary Figure 4c). *Atp2a2* encodes SERCA2a, an adaptive SR/ER-localized calcium ATPase previously shown to be ATF6-inducible in the heart^25^ and to improve contractility in heart failure patients^26^. None of the **147** dosing protocols resulted in pathological remodeling of the heart (Supplementary Figure 4d; Supplementary Table 2), cardiotoxicity, as evidenced by no increased plasma cTnI (Supplementary Figure 4e) or cardiac pathology-associated genes, such as *Nppa*, *Nppb*, *Col1a1*, or *Myh7* (Supplementary Figure 4f). Furthermore, no apparent deficits were observed in any of the experiments upon inspection of the liver or kidneys when steatosis and glomerular filtration rate were assessed by hepatic triglyceride accumulation and creatinine clearance, respectively (Supplementary Figure 4g, h). These results indicate a time course of gene induction and no toxic side effects of **147** that support the efficacy of **147** when administered in the setting of an I/R injury, either as a single dose or as part of a serial dosing regimen.

### 147 protects the heart from I/R injury in vivo

**Fig. 7:**
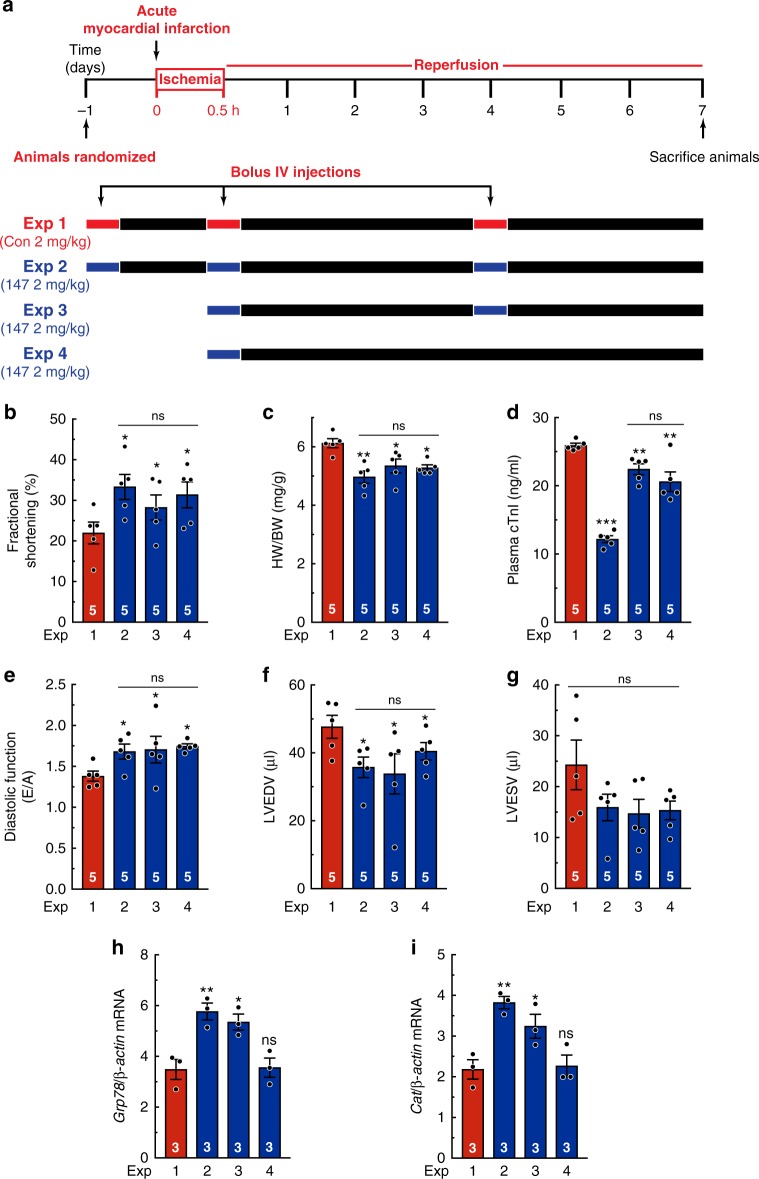
**147** improves cardiac performance 7 days post AMI. **a** Experimental design for testing the effects of **147** in the hearts of mice subjected to 30 min of myocardial infarction, and then examined 7 days after the initiation of reperfusion. Red bars depict the bolus administration of the control compound, while blue bars depict the bolus administration of **147**. **b**, **f**, **g** Echocardiographic parameters of fractional shortening (**b**), LV end diastolic volume (LVEDV) (**f**), and LV end systolic volume (LVESV) (**g**) (*n* = 5). Detailed analyses of echocardiography parameters are in Extended Data Table 3. **c** Ratio of heart weight to body weight (*n* = 5). **d** Plasma cTnI (*n* = 5). **e** Diastolic function as determined by pulse-wave Doppler (PW) technique to analyze E and A waves (*n* = 5). **h**, **i** qPCR for *Grp78* (**h**) or *Cat* (**i**) in LV of mice from indicated experiments at culmination of study (*n* = 3). Data are represented as mean ± s.e.m. Two-group comparisons were performed using Student’s two-tailed *t* test, and all multiple group comparisons were performed using a one-way ANOVA with a Newman–Keuls post hoc analysis. *P *≤* 0.05, **P *≤* 0.01, and ***P *≤* 0.001; ns not signficant

Next, the effects of **147** were examined in an in vivo model of I/R damage in the heart 7 days after reperfusion (Fig. 7a). In Experiments 1 and 2, the negative control compound or **147**, respectively, were administered 24 hours prior to AMI, with a second dose at reperfusion and a third dose 4 days later. In Experiment 3, **147** was administered at reperfusion and again 4 days later. In Experiment 4, **147** was administered only one time, at reperfusion. Given the transient nature of **147**, we designed our multiple-dose strategy so that it mimics a therapeutic approach used for treating AMI patients as soon as possible after the infarction, to mitigate the initial reperfusion damage to the heart, as well as days later to ameliorate the detrimental effects of continued expansion of infarct damage and cardiac remodeling in the infarct and infarct border zones on heart pump function. Strikingly, cardiac performance was preserved to similar extents in all experiments involving **147** (Fig. 7b), as was the ability of **147** to reduce cardiac hypertrophy, which is a pathological response to I/R in this model (Fig. 7c). **147** decreased plasma cTnI in all of the experiments, though somewhat less so in Experiments 3 and 4 (Fig. 7d). Importantly, **147** preserved diastolic cardiac function and left ventricular volumes in all of the experiments (Fig. 7e–g; Supplementary Table 3), showing that **147** impeded the progression toward heart failure. In Experiments 2 and 3, the beneficial structural and functional effects were accompanied by increased expression of the ATF6-regulated genes, *Grp78* and *Cat* (Fig. 7h–i; Supplementary Figure 5a), but not *Erdj4* and *Atf4* (Supplementary Figure 5b, c). However, in Experiment 4, the levels of *Grp78* and *Cat* were comparable to control-treated animals, as expected, given the transient nature of **147**-mediated gene induction seen in a previous experiment (see Fig. 6). Moreover, I/R induced cardiac pathology genes (Supplementary Figure 5d, Sham vs. Experiment 1), as expected; however, these effects were blunted by **147** (Supplementary Figure 5d, Experiments 2–4). In addition, decreased levels of pro-apoptotic cleaved caspase-3 were seen in Experiments 2–4 (Supplementary Figure 5e), indicating that **147** protected myocytes from apoptosis during I/R. Thus, pharmacologic ATF6 activation at reperfusion ameliorated pathologic cardiac dysfunction in response to I/R injury.

### 147 is beneficial in a wide range of disease models in vivo

**Fig. 8:**
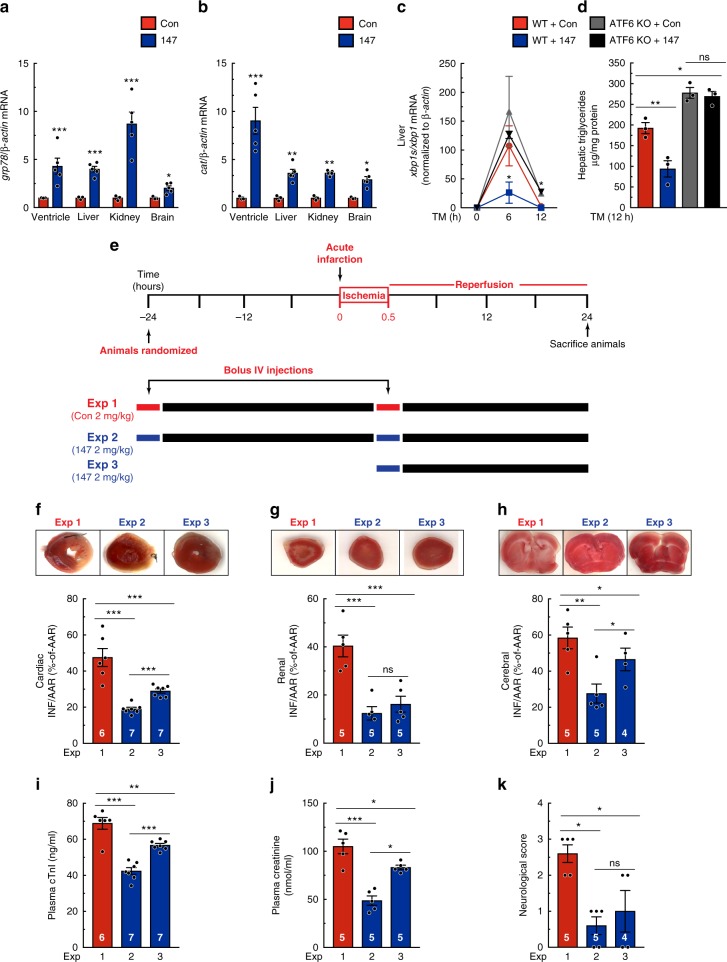
**147** exerts widespread protection in multiple organs. **a**, **b** qPCR for *Grp78* (**a**) or *Cat* (**b**) in left ventricular, liver, kidney, and brain extracts from WT mice 24 hours post treatment with control or **147** (n = 3). **c** Ratio of transcript levels of *Xbp1s* to *Xbp1* as determined by qPCR in liver extracts from WT or ATF6 KO mice 24 hours post treatment with control or **147** and then treated with 2 mg/kg of TM for designated periods of time (*n* = 3). **d** Triglyceride levels in liver extracts from WT or ATF6 KO mice 24 hours post treatment with control or **147** and then treated with 2 mg/kg of TM for 12 hours  (*n* = 3). **e** Experimental design for testing the effects of **147** in the hearts of mice subjected to 30 min of myocardial infarction, and then examined 24 hours after the initiation of reperfusion. Red bars depict the bolus administration of the control compound, while blue bars depict the bolus administration of **147**. **f**–**h** Relative infarct sizes in the heart (**f**) (*n* = 6–7 for each experiment, as shown), kidney (**g**), and brain (**h**) (*n* = 4–5 for each experiment, as shown) of male mice 24 hours after reperfusion. **i**–**k** Plasma cTnI (**i**) (*n* = 6–7 for each experiment, as shown), plasma creatinine (**j**), and neurological score based on the Bederson system of behavioral patterns post-cerebral ischemic injury of male mice 24 hours after reperfusion of respective injury models (*n* = 4–5 for each experiment, as shown). Data are represented as mean ± s.e.m. Two-group comparisons were performed using Student’s two-tailed *t* test, and all multiple group comparisons were performed using a one-way ANOVA with a Newman–Keuls post hoc analysis. **P *≤* 0.01, ***P *≤* 0.001; ns not significant

Next, we examined the effects of **147** following 24 hours of administration, an important time at which AMI patients are often treated by coronary angioplasty. Additionally, since ATF6 is expressed in all cells, we posited that it might be effective in tissues in addition to the heart. Accordingly, in addition to the heart, we determined the effects of **147** in the liver, kidney, and brain. **147** activated ATF6 target genes in all four of the tissues, as evidenced by significant increases in of *Grp78* and *Cat* (Fig. 8a, b), although the magnitude of the responses varied somewhat between tissues. The functionality of **147**-mediated activation of ATF6 in the liver was evident in that it significantly reduced ER protein misfolding, measured by XBP1 splicing, in mice that had been injected with TM; this beneficial effect was lost upon genetic deletion of *Atf6* (Fig. 8c). Additional evidence of the functionality of **147** in the liver was evident in its ability to reduce hepatic triglycerides, which are a hallmark of hepatic steatosis, demonstrating improved ER proteostasis in the liver (Fig. 8d, blue); this beneficial effect of **147** was also lost upon deletion of ATF6 (Fig. 8d, black).

Next, to examine the functional effects of **147** in the various tissues, the control compound or **147** were administered, as shown in Fig. 8e, and the effects were examined on tissue damage in the heart via the acute I/R model, the kidney via transient unilateral renal portal system occlusion, and in the brain via transient unilateral middle cerebral artery (MCA) occlusion. Throughout the studies, the surgeon and the data analyst were blinded to the animal assignments, which were predetermined by a separate investigator. Remarkably, even when administered only at the time of reperfusion, **147** significantly decreased infarct sizes in all three tissues when measured 24 hours after I/R (Fig. 8f–h; Supplementary Figure 6a). Moreover, **147** decreased plasma cTnI and creatinine, which are biomarkers of cardiac and kidney damage, respectively, and it improved behavioral indicators of post-ischemic neurological damage (Fig. 8i–k). As expected, since 24 hours after reperfusion is too short for structural remodeling, there were no observable deficits on cardiac performance, chamber size, or pathological hypertrophy, as monitored by echocardiography (Supplementary Table 4). As further proof of concept, this experiment was replicated in female mice and, again, both Experiments 2 and 3 conferred protection as evidenced by reduced infarct sizes and plasma cTnI (Supplementary Figure 6b, c). Importantly, these beneficial effects of **147** in response to myocardial acute I/R were not seen in ATF6 cKO mice, further emphasizing that **147**-mediated protection of the heart required ATF6 activation (Supplementary Figure 6d, e). Interestingly, the beneficial effects of **147** were also seen in a different AMI model induced by acute administration of the β-adrenergic receptor agonist, isoproterenol, which is known to cause widespread oxidative damage and cardiac myocyte death in mice at this dose (Supplementary Figure 6f–h).

Thus, when administered at the time of injury, **147** protected a wide range of tissues from I/R damage, emphasizing the broad spectrum of potential applications for this compound as a transcriptional regulator of the ATF6 arm of the UPR and subsequent reprogramming of proteostasis, in vivo.

## Discussion

**Fig. 9:**
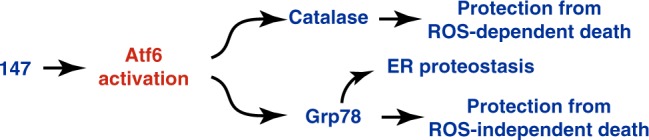
**147** mediates both ROS-dependent and ROS-independent protection globally. Proposed mechanism whereby 147 confers widespread global protection within the cell through activation of ATF6 to act both in a canonical and non-canonical manner to protect against ROS-dependent and ROS-independent challenges to proteostasis

After an AMI, upon reconstituting blood flow, reperfusion damage begins almost immediately and continues for at least 3 days^27^. The initial reperfusion damage is thought to be due ROS generation by mitochondria in the myocardium, while the longer term damage may be due to multiple mechanisms, including continued ROS generation by the infiltration of inflammatory cells into the infarct zone^13,28^. Therefore, an effective therapy for AMI should function over a timeframe spanning at least 3 days. While a number of potential therapies that act acutely to minimize reperfusion damage have been tested, many of them have failed to move through the drug development process and there is still no clinically available intervention^15^. At the outset of the current study, we posited that this might be because most of the previous therapeutics function only during the initial stages of reperfusion, losing efficacy in the ensuing days. Furthermore, many of the initial trials performed in small animals have not tested therapies at times that accurately mimic typical clinical interventions (i.e., during coronary angioplasty) and have not adhered to the Food and Drug Administration's (FDA’s) Good Laboratory Practices. Accordingly, in addition to addressing these points in the design of our animal experiments here, we examined the therapeutic function after both 1 and 7 days of reperfusion. We also set out to develop a therapeutic approach that would exert beneficial effects through multiple mechanisms in various cellular locations, which we felt would broaden the potential utility to include different tissues and widen the scope to multiple proteostasis-based pathologies. In this regard, we focused on ATF6, since it adaptively reprograms ER proteostasis by inducing a wide range of protective response genes that encode proteins, such as catalase and grp78, which act to mitigate ROS-induced damage, as well as emending ROS-independent proteostasis pathways, respectively (Fig. 9). Using this strategy, we found that selective pharmacologic activation of only the ATF6 arm of the UPR with **147** in mice acted within 1 day to reduce reperfusion damage in the heart. Moreover, we found that **147** acted after 7 days to preserve cardiac function. This timing of these beneficial effects is consistent with the timing of adaptive ATF6 target gene induction and the reperfusion damage that takes place over this same time frame, although we cannot rule out an effect of **147** on reducing damage during ischemia. In addition to demonstrating its efficacy in the heart, we found that **147** protected the liver in a mouse model of dysregulated hepatic proteostasis, and it protected the kidneys and brain in models of renal and cerebral I/R damage. These findings, together with a recent report showing that **147** enhances the differentiation of human embryonic stem cells^29^, support the broad therapeutic potential of pharmacologic activation of ATF6 for treating a wide range of proteostasis-based pathologies in various tissues.

In terms of its suitability as a pharmacologic agent, **147** exhibits many desirable properties. For example, **147** is highly specific, serving as the first example of a compound that selectively activates only one arm of the UPR, ATF6, which is well known for exerting mainly beneficial effects in many different cell types. **147** is highly efficacious in vivo, functioning at a dose similar to many other cardiovascular drugs and has the capacity to cross the blood–brain barrier. Moreover, **147** does not exhibit any apparent toxicity or deleterious off-target effects in vivo. Both the efficacy and tolerance of **147** can be attributed in large part to the high-stringency, cell-based transcriptional profiling that was done in the initial screening to ensure that **147** specifically activates only the ATF6 arm of the UPR, instead of global UPR activation^20^. The relatively transient activation of ATF6 by **147** in vivo is also potentially advantageous, since many stress-signaling pathways, including the UPR, can be beneficial initially, but damaging upon chronic activation^30^. Since I/R only partially activates ATF6, the remaining inactive ATF6 provides a therapeutic reserve for **147** to activate, allowing it to boost adaptive ATF6 signaling pathways in multiple tissues, in vivo. Remarkably, we found that **147** exerted beneficial effects in the hearts of mice that were not subjected to any injury maneuvers, underscoring the safety, and perhaps even benefits of the compound in healthy tissues. Thus, while future pharmacokinetic and toxicology studies will address further details of **147** action, it is clear from the results presented here that **147** is easily administered, well tolerated, acts quickly, boosts an endogenous adaptive transcriptional stress-signaling pathway, and has no apparent off-target or untoward effects, all of which are attributes of an excellent candidate for therapeutic development.

Impaired proteostasis contributes to numerous pathologies and even impacts aging^31^. Thus, global improvement of proteome quality through pharmacologic activation of defined transcriptional regulators of proteostasis should ameliorate a broad range of proteostasis-based diseases. Recent findings showing that the sphere of influence of the UPR, in particular, the ATF6 arm of the UPR, extends well beyond the ER to reprogram proteostasis in many cellular locations^10^, support the potential broad spectrum of impact of pharmacologic compounds, like **147**. The results presented here provide proof of principle that this type of pharmacologic correction can be achieved with well-characterized compounds, such as **147,** that selectively activate a specific protective aspect of UPR signaling.

## Methods

### Laboratory animals

The research reported in this article complies with all relevant ethical regulations and has been reviewed and approved by the San Diego State University Institutional Animal Care and Use Committee (IACUC), and conforms to the Guide for the Care and Use of Laboratory Animals published by the National Research Council. ATF6-floxed mice were a generous gift from Gokhan S. Hotamisligil. Briefly, ATF6-floxed mice were generated with a targeting construct flanking exons 8 and 9 of ATF6 with LoxP sequences on a C57B/6J background, as previously described^32^. For preclinical efficacy testing of experimental compounds, WT 10-week-old male or female C57B/6J mice were used (The Jackson Laboratory, Bar Harbor, ME, USA). For some experiments, we determined the number of animals to use based on a predictive power analysis to achieve 5% error and 80% power, or using the resource equation method^33^. In other experiments, the number of animals to use were determined practically, based on previous experiments designed to determine, for example, surgery mortality rates and the approximate magnitude of changes in the measured parameters. This was the case in experiments using ATF6 cKO mice. Our previous experiments showed that the variation in infarct size between littermates post in vivo I/R surgeries was low, amounting to <5%^10^. All animal work was performed at the same time of the circadian rhythm typical of animals housed on a 12-h light–dark cycle with ad libitum feeding. All studies in which compound **147** was administered to mice were conducted such that the surgeon and data analyst were blinded to the group assignments. Prior to all experiments, animals were assigned codes by one investigator, while investigator #2 was blinded to animal codes and nature of the treatments, for example, control vs. compound **147**, performed the surgeries and echocardiographic analysis. Investigator #3 analyzed the areas at risk and infarct regions for all cardiac, renal, and cerebral I/R injury models; as with investigator #2, this investigator was also blinded to the animal codes and treatments. Animals were not decoded until after all surgical, functional, and histological analyses were fully analyzed and relevant statistical assessments had been calculated for all parameters measured. For all animal experiments involving cKO of ATF6, ATF6-floxed littermates were randomly assigned to receive AAV-control or AAV-Cre (1:1 ratio) to minimize mouse-to-mouse variability. Animals involved in I/R experiments involving administration of either the control compound or compound **147**, WT 10-week-old male or female C57B/6J littermates. Consistency and, therefore, minimal variability of infarct sizes following ex vivo and in vivo I/R studies was ensured through blinded measurements of areas at risk relative to total left ventricular areas, as described above. As a result, we observe a variation in area at risk/left ventricle area (AAR/LV) within experimental groups of <5%. For ex vivo I/R studies, mechanical error and variability were maintained as low as possible by minimizing the time between animal sacrifice and initiation of retroperfusion; our criteria is that this process must take <60 s. We found that this results in a relatively rapid progression to equilibration of heart function during ex vivo perfusion; our criteria for reaching equilibration of LVDP is <15 min after initiation or retroperfusion on the Langendorff apparatus.

### Patient samples

Human heart explants were obtained from ventricular myocardium of patients with advanced ischemic heart failure. Control patient ventricular explants were obtained from non-failing donor hearts deemed unsuitable for transplantation for non-cardiac reasons. Samples were collected with informed consent and complying with all relevant ethical regulations as previously described^34^. All study procedures were approved by the University of Pennsylvania Hospital Institutional Review Board.

### Adeno-associated virus serotype 9

The plasmid encoding the human cardiac troponin T promoter driving Cre-recombinase was provided as a gift from Dr. Oliver Muller^35^. AAV9 preparation and injection were carried out as previously described^10,24^. Non-anesthetized 8-week-old ATF6-floxed mice were injected with 100 μl of AAV9-control or AAV9-cTnT-Cre containing 1 × 10^11^ viral particles via the lateral tail vein using a 27-guage syringe and housed for 2 weeks before either sacrifice or experimental initiation.

### Adenovirus

Construction of plasmid vectors encoding FLAG-tagged full-length inactive ATF6 [ATF6(1–670)], TCR-α-HA, and empty vector (AdV-Con) has been previously described^10,24^.

### Cardiomyocyte isolation, culture, and experimental design

Neonatal rat ventricular myocytes (NRVMs) were isolated via enzymatic digestion, purified by Percoll density gradient centrifugation, and maintained in Dulbecco’s modified Eagle’s medium (DMEM)/F12 supplemented with 10% fetal bovine serum (FBS) and antibiotics (100U/ml penicillin and 100 µg/ml streptomycin) on plastic culture plates that had been pre-treated with 5 µg/ml fibronectin, as previously described^10,24^. For all NRVM experiments, plating density was maintained at 4.5 × 10^5^ cells per well on 12-well plates. Adult mouse ventricular myocytes (AMVMs) were isolated from WT or ATF6 cKO mice 24 h after intravenous (IV) injection of control compound (2 mg/kg) or compound **147** (2 mg/kg). AMVM isolation was performed by cannulating the ascending aorta, followed by retroperfusion and collagenase digestion, as previously described^10^. For all experiments, AMVMs were plated at a density of 5.0 × 10^5^ cells per well on 24-well plates that had been pre-treated with laminin (10 µg/ml) and incubated in maintaining medium (MEM medium, 1× insulin-transferrin-selenium, 10 mM HEPES, 1.2 mM CaCl_2_, and 0.01% bovine serum albumin, 25 μM blebbistatin) for 16 h before initiating experiments as previously described^10^. Sixteen hours after plating, NRVMs and AMVMs were treated with control compound (10 µM), compound **147** (10 µM), or TM (10 µg/ml) for 24 h in DMEM/F12 supplemented with bovine serum albumin (BSA) (1 mg/ml) for NRVMs, or maintaining media for AMVMs. For in vitro I/R, ischemia was simulated by replacing all culture media with 0.5 ml of glucose-free DMEM containing 2% dialyzed FBS with either the control compound (10 µM) or compound **147** (10 µM), and then incubated at 0.1% O_2_ in a hypoxia chamber with an oxygen controller (ProO × P110 oxygen controller, Biospherix, Parish, NY, USA) for 8 or 3 h for NRVMs or AMVMs, respectively, as previously described^10^. Reperfusion was simulated by replacing culture media with DMEM/F12 supplemented with BSA (1 mg/ml) for NRVMs or maintaining media for AMVMs and incubating at 21% O_2_ for an additional 24 h. NRVM and AMVM reperfusion media were supplemented with control compound (10 µM) or compound **147** (10 µM) throughout the duration of the reperfusion period. Viability was determined as the number of calcein-AM-labeled NRVMs or rod-shaped calcein-AM-labeled AMVMs, using calcein-AM green (Thermo Fisher). Images were obtained with an IX70 fluorescence microscope (Olympus, Melville, NY, USA). The number of viable, calcein-AM green-positive cells were counted using ImageJ or Image-Pro Plus software (Medium Cybernetics, Rockville, MD, USA).

### siRNA transfection

Transfection of small interfering RNA (siRNA) into NRVMs was achieved using HiPerfect Transfection Reagent (Qiagen, Valencia, CA, USA) following the vendor’s protocol. Briefly, NRVM culture medium was replaced with DMEM/F12 supplemented with 0.5% FBS without antibiotics, 120 nM siRNA, and 1.25 µl HiPerfect/1 µl siRNA, and then incubated for 16 h, after which the culture medium was replaced with DMEM/F12 supplemented with BSA (1 mg/ml) for an additional 48 h. The sequence of siRNA targeting rat ATF6 was 5-GCUCUCUUUGUUGUUGCUUAGUGGA-3, the sequence targeting rat catalase was 5-GGAACCCAAUAGGAGAUAAACUUAA-3 (cat# CatRSS302058, Stealth siRNA, Thermo Fisher), and the sequence targeting rat grp78 was 5-AGUGUUGGAAGAUUCUGA-3 (cat# 4390771, Stealth siRNA, Thermo Fisher) as previously described^10^. A non-targeting sequence (cat# 12935300, Thermo Fisher) was used as a control siRNA.

### Immunoblot analysis

NRVMs were lysed and subjected to immunoblot analysis, as previously described^10^. In brief, cultures were lysed with VC lysis buffer made from 20 mM Tris-HCl (pH 7.5), 150 mM NaCl, 0.1% sodium dodecyl sulfate (SDS), 1% Triton X-100, protease inhibitor cocktail (Roche Diagnostics, Indianapolis, IN, USA), and phosphatase inhibitor cocktail (Roche Diagnostics). Samples comprising 10 µg of protein were mixed with Laemmli sample buffer, boiled, then subjected to SDS-polyacrylamide gel electrophoresis (SDS-PAGE), followed by transfer onto PVDF membranes for immunoblotting. Full-length Atf6 (p90) was detected with an antibody from SAB Signalway Antibody (1:1000, cat# 32008, College Park, MD, USA), while active Atf6 (p50) was detected with an antibody from Proteintech (1:1000, cat# 24169-1-AP, Rosemont, IL, USA). Other antibodies used include: anti-KDEL antibody (1:8000, cat# ADI-SPA-827, Enzo Life Sciences, Farmingdale, NY, USA), which was used to detect GRP78, anti-catalase (1:1000, cat# ab16731, Abcam), anti-IRE1 (1:500, cat# sc-390960, Santa Cruz), anti-XBP1s (1:1000, cat# 619502, BioLegend, San Diego, CA, USA), anti-phospho-PERK (1:1000, cat# 3179, Cell Signaling), anti-PERK (1:1000, cat# 3192, Cell Signaling), anti-Anp (1:4000, cat# T-4014, Peninsula), anti-Gapdh (1:25,000, cat# G109a, Fitzgerald Industries International Inc.), HA-probe F-7 (Santa Cruz, SC-7392; 1:1000), and anti-FLAG (1:3000, cat#F1804, Sigma-Aldrich, St. Louis, MO, USA). The oxidation state of ATF6 in NRVMs treated with **147** was analyzed by gel-shift essentially as previously described^32^. Briefly, cells were lysed in low-stringency lysis buffer comprising 20 mM Tris-HCl (pH 7.5), 150 mM NaCl, 1% Triton X-100, protease inhibitor cocktail (Roche Diagnostics, Indianapolis, IN, USA) and phosphatase inhibitor cocktail (Roche Diagnostics) and 20 µM 4-acetamido-4ʹ-maleimidylstilbene-2,2ʹ-disulfonic acid, disodium salt (AMS) (Thermo Fisher, cat# A485). AMS binds covalently to reduced thiols, typically on cysteine residues, and increases their molecular mass in SDS-PAGE. Thus, proteins that exhibit an upward shift when analyzed under non-reducing conditions compared to reducing are considered to have reduced thiols.

### Quantitative reverse transcription PCR

Total RNA was extracted from left ventricular extract using the RNeasy Mini Kit (Qiagen) as previously described^10^. All qPCR probes were obtained from Integrated DNA Technologies and primer sequences are listed in Supplementary Table 5.

### Immunocytochemistry and immunohistochemistry

NRVMs and AMVMs were plated on fibronectin- and laminin-coated glass chamber slides, respectively, as previously described^10^. In brief, cells were fixed with 4% paraformaldehyde, followed by permeabilization with 0.5% Triton X. Adult mouse hearts were paraffin-embedded after fixation in neutral-buffered 10% formalin via abdominal aorta retroperfusion as previously described^10^. The infarct border zone was imaged in hearts subjected to surgical I/R. The infarct border zone was identified as an area that stained positively for the cardiac muscle protein tropomyosin that was adjacent to an area that did not stain for tropomysin (infarct zone) due to the absence of viable myocytes. The left ventricular free wall was imaged in sham and non-injured hearts. Primary antibodies used were anti-α-actinin (1:200, cat# A7811, Sigma-Aldrich), anti-tropomyosin (1:200, cat# T9283, Sigma-Aldrich), anti-GRP78 (C-20, 1:30, cat# SC-1051, Santa Cruz), anti-catalase (1:100, Abcam), anti-ATF6 (targeting to N terminus of ATF6, 1:50, cat# sc-14250, Santa Cruz), and anti-cleaved caspase-3 (1:100, cat# D175, Cell Signaling). Slides were incubated with appropriate fluorophore-conjugated secondary antibodies (1:100, Jackson ImmunoResearch Laboratories, West Grove, PA, USA) followed by nuclei counter-stain Topro-3 (1:2000, Thermo Fisher). Images were obtained using laser scanning confocal microscopy on an LSM 710 confocal laser scanning microscope (Carl Zeiss, Oberkochen, Germany).

### ERAD assay

ERAD was determined using a C-terminal HA-tagged version of the model chronic misfolded substrate, TCR-*α*-HA, as previously described^24^.

### Luciferase secretion assay

Secretory capacity of cardiac myocytes was determined essentially as described^36^. Briefly, NRVMs were co-transfected with pcDNA plasmid as well as p-SV-*β*-galactosidase control vector and pCMV-GLuc plasmid (NEB, N8081S) using FuGENE6 (2 µg complementary DNA (cDNA), 2:1, FuGENE:cDNA).

### Chromatin immunoprecipitation

Chromatin immunoprecipitation (ChIP) assays were performed essentially as previously described^10^. Briefly, AdV-FLAG-ATF6(1–670)-infected NRVMs were treated with fixing buffer (50 mM HEPES-KOH, pH 7.5, 100 mM NaCl, 1 mM EDTA, 0.5 mM EGTA, and 1% formaldehyde) for 10 min, quenched with 125 mM glycine, and scraped into ice-cold phosphate-buffered saline (PBS). Cells were centrifuged, resuspended in lysis buffer (50 mM HEPES, pH 7.9, 140 mM NaCl, 1 mM EDTA, 10% glycerol, 0.5% NP-40, 0.25% Triton X-100, and protease inhibitor cocktail), and incubated on ice for 10 min. After centrifugation at 1800 *×* *g* for 10 min, the pellets were washed with buffer containing 10 mM Tris, pH 8.1, 200 mM NaCl, 1 mM EDTA, and 0.5 mM EGTA, resuspended in shearing buffer (0.1% SDS, 1 mM EDTA, and 10 mM Tris, pH 8.1), and then transferred to microTUBEs (Covaris, Woburn, MA, USA). Chromatin was sheared by sonication for 15 min using an M220 focused ultrasonicator (Covaris). Triton X-100 and NaCl were added to the final concentration of 1% Triton and 150 mM NaCl followed by centrifugation at 16,000 × *g* for 10 min. Immunoprecipitation was performed by incubated 140 μl of sheared chromatin with 5 μg of anti-FLAG antibody (cat# F1804, Sigma-Aldrich) and 260 μl of immunoprecipitation buffer (0.1% SDS, 1 mM EDTA, 10 mM Tris, pH 8.1, 1% Triton X-100, and 150 mM NaCl) at 4 °C overnight. Protein A/G magnetic beads (5 μl, BcMag, Bioclone, San Diego, CA, USA) were added to the mixtures and incubated at 4 °C for 1.5 h. Magnetic beads were sequentially washed with low salt wash buffer (0.1% SDS, 1% Triton X-100, 2 mM EDTA, 20 mM HEPES-KOH, pH 7.9, and 150 mM NaCl), high salt wash buffer with 500 mM NaCl, LiCl wash buffer (100 mM Tris-HCl, pH 7.5, 0.5 M LiCl, 1% NP-40, and 1% deoxycholate acid), and TE buffer (10 mM Tris-HCl, pH 8.0, and 0.1 mM EDTA). Immune complexes were eluted by incubating beads with proteinase K digestion buffer (20 mM HEPES, pH 7.9, 1 mM EDTA, 0.5% SDS, and 0.4 mg/ml proteinase K) at 50 °C for 15 min. Formaldehyde cross-linking was reversed by incubating with 0.3 M NaCl and 0.3 mg/ml RNase A at 65 °C overnight. Samples were further incubated with 550 μg/ml proteinase K at 50 °C for 1 h. DNA was purified using NucleoSpin Gel and PCR Clean-up Kit (Macherey-Nagel, Bethlehem, PA, USA) and eluted by 30 μl of water. Two microliters of DNA was used for qRT-PCR analysis with primers targeting rat Hspa5 (5ʹ-GGTGGCATGAACCAACCAG-3ʹ and 5ʹ-GCTTATATATCCTCCCCGC-3), rat Cat ERSE-1 (5ʹ-CTACCCACCAATTAGTACCAAATAA-3ʹ and 5ʹ-AGAAGGGACAGGATTGGAAG-3ʹ), rat Cat ERSE-2 (5ʹ-CACATTCTAGGGACAGTGTAGATG-3ʹ and 5ʹ-ACCTTGATTATGGGCTGTGG-3ʹ), rat Pdia6 ERSE (5ʹ-CACATGAGCGAAATCCACAGA-3ʹ and 5ʹ-ACTAGTCGAGCCATGCTGAT-3ʹ), rat HO-1 (5ʹ-GGGCTACTCCCGTCTTCCTG-3ʹ and 5ʹ-CCTTTCCAGAACCCTCTACTCTACTC-3ʹ), or rat Gapdh (5ʹ-ATGCGGTTTCTAGGTTCACG-3ʹ and 5ʹ-ATGTTTTCTGGGGTGCAAAG-3ʹ). Pdia6 served as a positive control for a known ATF6 target gene in cardiac myocytes, while HO-1 and Gapdh served as negative controls as previously described^37^. ChIP signals obtained from the qRT-PCR were normalized to the input DNA.

### Ex vivo I/R

Hearts from WT or ATF6 cKO mice that had previously received 2 mg/kg IV administration of control compound or compound **147** were rapidly excised and cannulated via the ascending aorta and subjected to global I/R, as previously described^38^. Here, the hearts were subjected to 20 min global no-flow ischemia followed by reperfusion for 1 h. LVDP was measured using a pressure sensor balloon placed into the left ventricle and analyzed using the Powerlab software (ADInstruments, Colorado Springs, CO, USA).

### In vivo myocardial I/R

Surgical myocardial I/R was performed as previously described^10^. Briefly, mice were anesthetized with 2% isoflurane and a thoracotomy was performed to isolate the heart, after which the left anterior descending coronary artery (LAD) was ligated with a 6-0 Prolene suture for 30 min, followed by suture removal and either 24 h or 7 days of reperfusion. Regional ischemia was confirmed by visual inspection of the discoloration of the myocardium distal of the ligation, which is characteristic of impaired blood flow. Animals assigned as shams underwent the thoracotomy surgical procedure, but were not subjected to LAD ligation. Animals were randomly assigned to experimental groups prior to outset of the experiment by a single investigator, while the surgeon and data analyst were blinded to group assignments. Animals designated to receive either control compound or compound **147** at the time of reperfusion received 2 mg/kg of respective compounds via IV injection 5 min prior to release of the ligation. Twenty-four hours after reperfusion, 1% of Evans Blue was injected apically to determine the AAR. Hearts were harvested and 1-mm sections of the hearts were stained with 1% 2,3,5-triphenyltetrazolium chloride (TTC) to measure the infarcted area (INF) as previously described^36^. The AAR, INF, and LV from digitized images of heart sections were analyzed using the ImageJ software. For all infarct data presented, respective AAR was normalized to total LV area and all compared experiments displayed the same AAR/LV ratios. A separate investigator analyzed the AAR, INF, and LV and was blinded to the animal assignments. Just prior to sacrifice, post I/R, animals were anesthetized and 0.5 ml of arterial blood were obtained via inferior vena cava puncture as previously described^33^. Blood was placed in heparin- and EDTA-coated vacutainer (BD Vacutainer) and centrifuged at 1800 × *g* for 10 min and plasma samples were analyzed for cTnI with a Mouse cTnI High-Sensitivity ELISA Kit (Life Diagnostics Inc.).

### In vivo renal I/R

Surgical renal I/R was performed as previously described^39^. Briefly, mice were anesthetized with 2% isoflurane and a 3 cm incision was made upon the abdominal midline and the abdominal cavity entered via an incision along the linea alba. The right kidney was visualized and separated from surrounding connective tissue. The right ureter and right renal portal system was permanently ligated and a right unilateral nephrectomy performed. Subsequently, the left kidney was visualized and separated from surrounding connective tissue. A Bulldog Clamp (Fine Science Tools, Foster City, CA, USA) was applied temporarily ligating the left renal portal system for a period of 30 min. Global ischemia was confirmed by visual inspection of the discoloration of the kidney of the ligation, which is characteristic of impaired blood flow. After that duration, the Bulldog Clamp was removed and the abdomen closed with instant tissue adhesive. Animals were randomly assigned to experimental groups prior to outset of the experiment by a single investigator, while the data analyst was blinded to experiment assignments. Animals designated to receive either control compound or compound **147** at the time of reperfusion received 2 mg/kg of respective compounds via IV injection 5 min prior to release of the ligation. Twenty-four hours after reperfusion, kidneys were harvested and 1-mm sections of the kidneys were stained with 1% TTC to measure the INF as previously described^39^. Just prior to sacrifice, post I/R, animals were anesthetized and 0.5 ml of arterial blood were obtained via inferior vena cava puncture as previously described^40^. Blood was placed in heparin- and EDTA-coated vacutainer (BD Vacutainer) and centrifuged at 1800 × *g* for 10 min and plasma samples were analyzed for creatinine as a measure of glomerular filtration rate and renal functional output with a Creatinine Assay Kit (Abcam).

### In vivo cerebral I/R

Surgical cerebral I/R was performed as previously described^11^. Briefly, mice were anesthetized with 2% isoflurane and a 3 cm incision was made along the midline of the ventral surface of the neck along the left side of the trachea. The left external and internal carotid arteries were visualized and dissected from surrounding connective tissue without disturbing tangential nerves. An 8-0 catheter filament 10 mm in length (Doccol Corporation) was to be inserted into the MCA via the internal carotid artery. This occluded blood flow to the MCA and was left in position for a period of 30 min. After that duration, the catheter was removed and the neck closed with instant tissue adhesive. Animals were randomly assigned to experimental groups prior to outset of the experiment by a single investigator, while the data analyst was blinded to experimental assignments. Animals designated to receive either control compound or compound **147** at the time of reperfusion received 2 mg/kg of respective compounds via IV injection 5 min prior to release of the ligation. Twenty-four hours after reperfusion, brains were harvested and 1-mm sections of the brains were stained with 1% TTC to measure the INF as previously described^41^. Just prior to sacrifice, animals were assigned a behavioral score to assess the severity of neurological function and deficit as a result of the cerebral ischemia. The scoring was performed based on the Bederson Neurological Examination Grading System^42^, where a grade of 0 corresponded to a normal function with no observable deficit, grade 1 to a moderate deficit with animals exhibiting forearm flexion, grade 2 to a severe deficit with decreased resistance to a lateral push when suspended by the tail and lethargy, and grade 3 to a severe deficit with extreme lethargy and circling behavior in the cage.

### Hepatic triglyceride assay

Hepatic triglyceride assay was performed as previously described^43^. Briefly, livers were harvested and 10 mg extracts were homogenized and analyzed for triglyceride content using the EnzyChrom Triglyceride Assay Kit (BioAssay Systems).

### Transthoracic echocardiography

Transthoracic echocardiography was performed using an ultrasound imaging system (Vevo 2100 System, Fujifilm VisualSonics, Toronto, ON, Canada) as described^24^. Diastolic function was determined as previously described^40^. Briefly, echocardiography coupled with pulse-wave Doppler was used to visualize transmitral flow velocities and were recorded by imaging the mitral orifice at the point of the mitral leaflets. Waveforms were recorded and analyzed for peak early- and late-diastolic transmitral flow velocities corresponding to E and A waves, respectively.

### Acute isoproterenol myocardial damage

Myocardial damage was induced by administering high-dose (200 mg/kg) isoproterenol via intraperitoneal injection in mice as previously described^40^.

### Malondialdehyde assay

Lipid peroxidation was determined by measuring the levels of malondialdehyde using a TBARS Assay Kit (Cayman Chemical, Ann Arbor, MI, USA) according to the manufacturer’s instructions as previously described^10^.

### In vivo experimental compound administration

Control compound and compound **147** were suspended to a final concentration of 0.2 mg/ml in 10% dimethyl sulfoxide. Mice were weighed prior to administration of compounds and, subsequently, non-anesthetized 10-week-old WT or ATF6 cKO mice were injected with ~250 µl of stock compounds via the lateral tail vein depending upon body mass to ensure accurate administration of 2 mg/kg. This dose was established in preliminary experiments with the control compound or compound **147** where it was shown to activate Atf6 in vivo; the prototypical UPR inducer, TM, which was also administered to mice at 2 mg/kg, as previously shown^44^ was used as a control. Since compound **147** and TM have similar molecular weights, this dose of **147** is near the molar equivalent of the typical dose of TM. It is relevant to note that for compound **147**, a dose of 2 mg/kg is similar to FDA-approved cardiovascular drugs, such as many angiotensin-converting enzyme inhibitors, which are used in small animal models at 2 mg/kg^45^.

### Statistics

For studies involving induction of myocardial damage, either through surgical I/R or isoproterenol administration, cohort sizes were based on a predictive power analysis to achieve 5% error and 80% power. All acute in vivo I/R studies in which compound **147** were conducted such that the surgeon and data analyst was blinded to the group assignments. Two-group comparisons were performed using Student’s two-tailed *t* test, and all multiple group comparisons were performed using a one-way analysis of variance (ANOVA) with a Newman–Keuls post hoc analysis. Data are represented as mean with all error bars indicating ± s.e.m. *P ≤ 0.05, **P ≤ 0.01, and ***P ≤ 0.001.

## Supplementary information

Supplementary Information

## Data Availability

The datasets generated during and/or analyzed during the current study are available from the corresponding author on reasonable request and a reporting summary is available as a supplementary file.

## References

[1] Hartl FU, Bracher A, Hayer-Hartl M (2011). Molecular chaperones in protein folding and proteostasis. Nature.

[2] Labbadia J, Morimoto RI (2015). The biology of proteostasis in aging and disease. Annu. Rev. Biochem..

[3] Das I (2015). Preventing proteostasis diseases by selective inhibition of a phosphatase regulatory subunit. Science.

[4] Roth DM, Balch WE (2011). Modeling general proteostasis: proteome balance in health and disease. Curr. Opin. Cell Biol..

[5] Walter P, Ron D (2011). The unfolded protein response: from stress pathway to homeostatic regulation. Science.

[6] Smith MH, Ploegh HL, Weissman JS (2011). Road to ruin: targeting proteins for degradation in the endoplasmic reticulum. Science.

[7] Chiang WC, Hiramatsu N, Messah C, Kroeger H, Lin JH (2012). Selective activation of ATF6 and PERK endoplasmic reticulum stress signaling pathways prevent mutant rhodopsin accumulation. Invest. Ophthalmol. Vis. Sci..

[8] Cooley CB (2014). Unfolded protein response activation reduces secretion and extracellular aggregation of amyloidogenic immunoglobulin light chain. Proc. Natl. Acad. Sci. USA.

[9] Martindale JJ (2006). Endoplasmic reticulum stress gene induction and protection from ischemia/reperfusion injury in the hearts of transgenic mice with a tamoxifen-regulated form of ATF6. Circ. Res..

[10] Jin JK (2017). ATF6 decreases myocardial ischemia/reperfusion damage and links ER stress and oxidative stress signaling pathways in the heart. Circ. Res..

[11] Yu Z (2017). Activation of the ATF6 branch of the unfolded protein response in neurons improves stroke outzcome. J. Cereb. Blood Flow Metab..

[12] Roth GA (2017). Global, regional, and national burden of cardiovascular diseases for 10 causes, 1990 to 2015. J. Am. Coll. Cardiol..

[13] Frangogiannis NG (2015). Pathophysiology of myocardial infarction. Compr. Physiol..

[14] Hausenloy DJ, Yellon DM (2007). The evolving story of “conditioning” to protect against acute myocardial ischaemia–reperfusion injury. Heart.

[15] Bulluck H, Yellon DM, Hausenloy DJ (2016). Reducing myocardial infarct size: challenges and future opportunities. Heart.

[16] Hausenloy DJ, Yellon DM (2016). Ischaemic conditioning and reperfusion injury. Nat. Rev. Cardiol..

[17] Kalogeris T, Baines CP, Krenz M, Korthuis RJ (2016). Ischemia/reperfusion. Compr. Physiol..

[18] Murphy E, Steenbergen C (2008). Mechanisms underlying acute protection from cardiac ischemia–reperfusion injury. Physiol. Rev..

[19] Yellon DM, Hausenloy DJ (2007). Myocardial reperfusion injury. N. Engl. J. Med..

[20] Plate, L. et al. Small molecule proteostasis regulators that reprogram the ER to reduce extracellular protein aggregation. *Elife* **5**, 10.7554/eLife.15550 (2016). 10.7554/eLife.15550 PMC4954754 27435961

[21] Wang J, Lee J, Liem D, Ping P (2017). HSPA5 gene encoding Hsp70 chaperone BiP in the endoplasmic reticulum. Gene.

[22] Kumar M (2016). Animal models of myocardial infarction: mainstay in clinical translation. Regul. Toxicol. Pharmacol..

[23] Dixon JA, Spinale FG (2010). Pathophysiology of myocardial injury and remodeling: implications for molecular imaging. J. Nucl. Med..

[24] Doroudgar S (2015). Hrd1 and ER-associated protein degradation, ERAD, are critical elements of the adaptive ER stress response in cardiac myocytes. Circ. Res..

[25] Thuerauf DJ (2001). Sarco/endoplasmic reticulum calcium ATPase-2 expression is regulated by ATF6 during the endoplasmic reticulum stress response: intracellular signaling of calcium stress in a cardiac myocyte model system. J. Biol. Chem..

[26] Gwathmey JK, Yerevanian A, Hajjar RJ (2013). Targeting sarcoplasmic reticulum calcium ATPase by gene therapy. Hum. Gene Ther..

[27] Baines CP (2011). How and when do myocytes die during ischemia and reperfusion: the late phase. J. Cardiovasc. Pharmacol. Ther..

[28] Hernandez-Resendiz S (2018). The role of redox dysregulation in the inflammatory response to acute myocardial ischaemia–reperfusion injury–adding fuel to the fire. Curr. Med. Chem..

[29] Kroeger H (2012). Induction of endoplasmic reticulum stress genes, BiP and chop, in genetic and environmental models of retinal degeneration. Invest. Ophthalmol. Vis. Sci..

[30] Ron D, Walter P (2007). Signal integration in the endoplasmic reticulum unfolded protein response. Nat. Rev. Mol. Cell. Biol..

[31] Martinez G, Duran-Aniotz C, Cabral-Miranda F, Vivar JP, Hetz C (2017). Endoplasmic reticulum proteostasis impairment in aging. Aging Cell.

[32] Engin F (2013). Restoration of the unfolded protein response in pancreatic beta cells protects mice against type 1 diabetes. Sci. Transl. Med..

[33] Charan J, Kantharia ND (2013). How to calculate sample size in animal studies?. J. Pharmacol. Pharmacother..

[34] Bedi KC (2016). Evidence for intramyocardial disruption of lipid metabolism and increased myocardial ketone utilization in advanced human heart failure. Circulation.

[35] Werfel S (2014). Rapid and highly efficient inducible cardiac gene knockout in adult mice using AAV-mediated expression of Cre recombinase. Cardiovasc. Res..

[36] Lynch JM (2012). A thrombospondin-dependent pathway for a protective ER stress response. Cell.

[37] Vekich JA, Belmont PJ, Thuerauf DJ, Glembotski CC (2012). Protein disulfide isomerase-associated 6 is an ATF6-inducible ER stress response protein that protects cardiac myocytes from ischemia/reperfusion-mediated cell death. J. Mol. Cell. Cardiol..

[38] Jin JK (2008). Localization of phosphorylated alphaB-crystallin to heart mitochondria during ischemia–reperfusion. Am. J. Physiol. Heart Circ. Physiol..

[39] Wei Q, Dong Z (2012). Mouse model of ischemic acute kidney injury: technical notes and tricks. Am. J. Physiol. Ren. Physiol..

[40] Wallner M (2016). Acute catecholamine exposure causes reversible myocyte injury without cardiac regeneration. Circ. Res..

[41] Xie M (2014). Histone deacetylase inhibition blunts ischemia/reperfusion injury by inducing cardiomyocyte autophagy. Circulation.

[42] Bederson JB (1986). Rat middle cerebral artery occlusion: evaluation of the model and development of a neurologic examination. Stroke.

[43] DeZwaan-McCabe D (2017). ER stress inhibits liver fatty acid oxidation while unmitigated stress leads to anorexia-induced lipolysis and both liver and kidney steatosis. Cell Rep..

[44] Wu J (2007). ATF6alpha optimizes long-term endoplasmic reticulum function to protect cells from chronic stress. Dev. Cell.

[45] Eckman EA (2006). Regulation of steady-state beta-amyloid levels in the brain by neprilysin and endothelin-converting enzyme but not angiotensin-converting enzyme. J. Biol. Chem..

