# Supplementary material for: Pharmacologic ATF6 activation confers global protection in widespread disease models by reprograming cellular proteostasis
Source: Nat Commun. 2019 Jan 14;10:187. doi: 10.1038/s41467-018-08129-2 (PMC6331617; doi:10.1038/s41467-018-08129-2)
Supplement: Supplementary file 1 — Supplementary Information [file 41467_2018_8129_MOESM1_ESM.pdf]

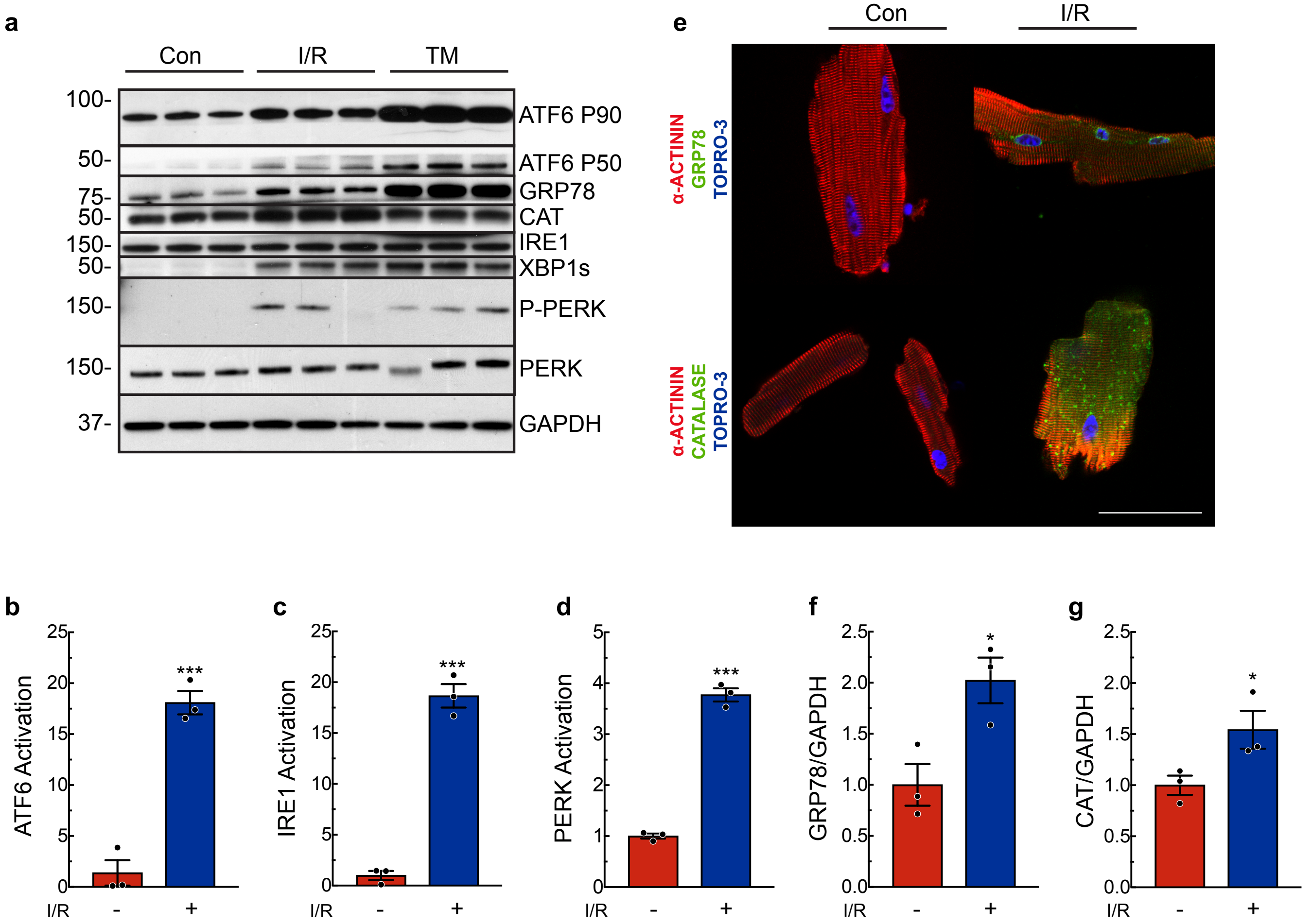

Supplementary Fig. 1 –I/R activates the UPR.

a, Immunoblots of neonatal rat ventricular myocytes (NRVM) for the proteins shown after I/R or tunicamycin (TM). **b-d,** Quantification of immunoblots from NRVM subjected to normoxia or I/R. ATF6, IRE1, and PERK activation are displayed as ratios of active fragment ATF6 (50kd), spliced-XBP1 and phospho-PERK relative to ATF6 (90kd), IRE1, and PERK, respectively (n=3). **e,** Immunocytofluorescence (ICF) for GRP78 or CAT (green), alpha-actinin (red) and nuclei (TOPRO-3) in isolated adult cardiomyocytes (AMVM) post-I/R. Scale bar represents 50μm. **f, g,** Quantification of immunoblots for Grp78 (**f**) or Cat (**g**) from NRVM subjected to normoxia or I/R. Data are represented as mean ± s.e.m. Two-group comparisons were performed using Student's two-tailed t-test, and all multiple group comparisons were performed using a one-way ANOVA with a Newman-Keuls post-hoc analysis. *P≤0.05, ***P≤0.001.

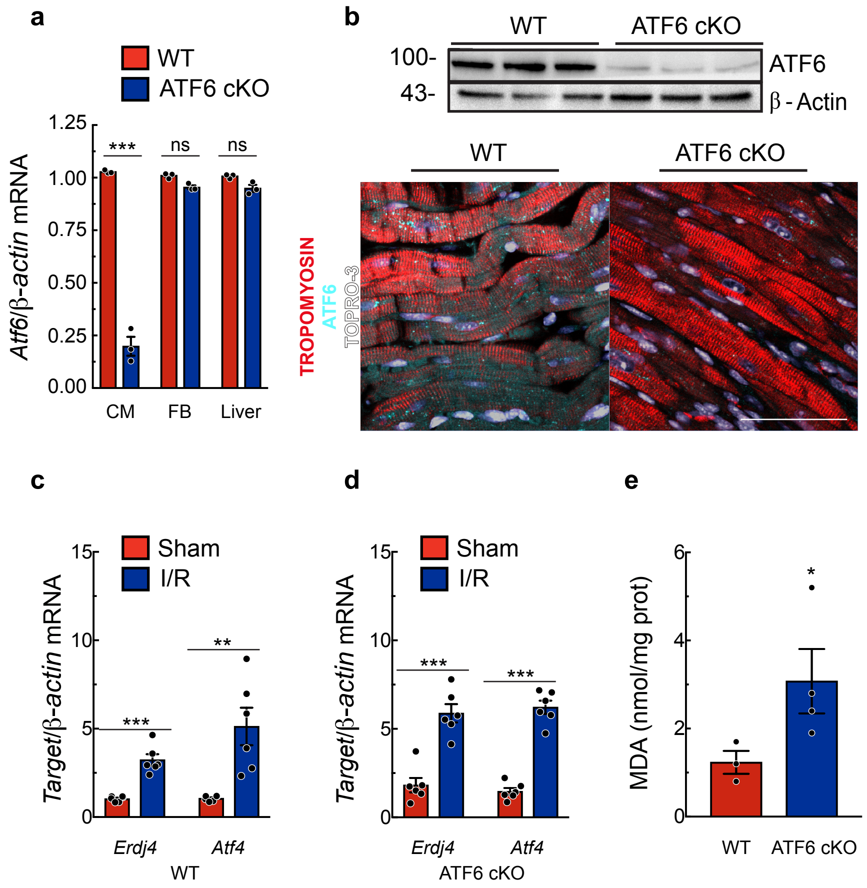

Supplementary Fig. 2 –Endogenous ATF6 is cardioprotective in a model of an acute AMI.
a, qPCR for atf6 in isolated adult mouse ventricular myocytes (AMVM), isolated cardiac fibroblasts, or liver extracts from WT (n=3) or ATF6 cKO (n=3) mice. **b,** Immunoblot for Atf6 and loading control, β-actin, and IHC staining for ATF6 (cyan), tropomyosin (red), and nuclei (TOPRO-3) in LV of WT or ATF6 cKO mice. Scale bar represents 50μm. **c, d,** qPCR for IRE1 downstream target, Erdj4, or PERK downstream target, Atf4 in the border zone of WT (**c**) (n=6) or ATF6 cKO (n=6) (**d**) hearts 24-hours after I/R. **e,** Malondialdehyde (MDA) in WT (n=3) and ATF6 cKO (n=3) mice 24-hours post-I/R. Data are represented as mean ± s.e.m. Two-group comparisons were performed using Student's two-tailed t-test, and all multiple group comparisons were performed using a one-way ANOVA with a Newman-Keuls post-hoc analysis. *P≤0.05, **P≤0.01, ***P≤0.001.

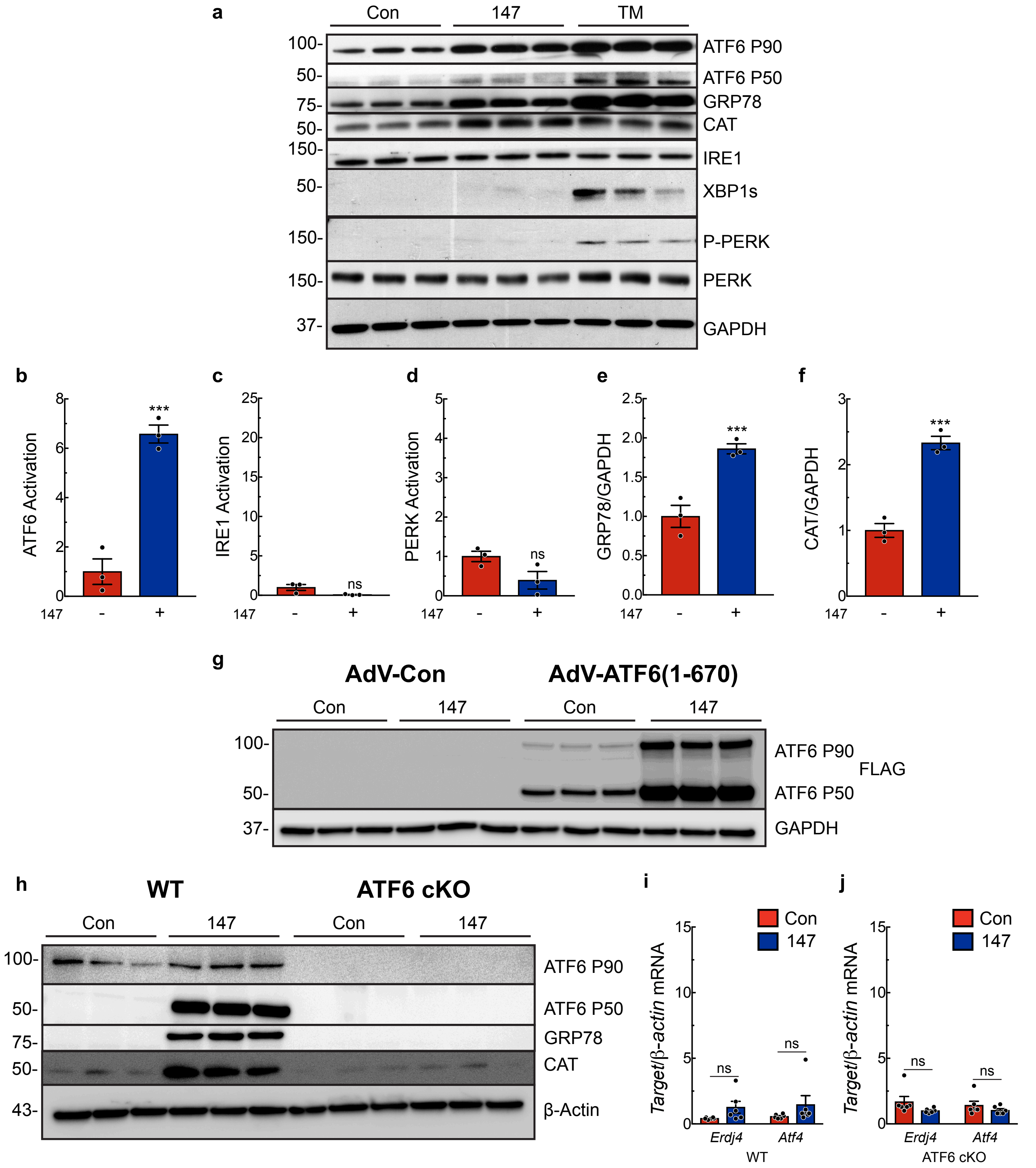

Supplementary Fig. 3 –147 is selectively activates ATF6.

a, Immunoblots of UPR target proteins from NRVM 24-hours after treatment with compound 147 or tunicamycin (TM). **b-f,** Quantification of immunoblots of NRVM treated with control or 147 (n=3). **g,** Immunoblot of NRVM infected with AdV encoding Flag-ATF6 (1-670) 24-hours after treatment with control or compound 147. Samples were performed in coordination with ChIP in Fig. 3e. **h,** Immunoblots of UPR target proteins from LV of WT (n=6) or ATF6 cKO (n=6) hearts 24-hours after treatment with control or 147. **i, j,** qPCR for Erdj4 or Atf4 in LV of WT (**i**) or ATF6 cKO (**j**) hearts 24-hours after treatment with control or 147. Data are represented as mean ± s.e.m. Two-group comparisons were performed using Student's two-tailed t-test, and all multiple group comparisons were performed using a one-way ANOVA with a Newman-Keuls post-hoc analysis. *P≤0.05, **P≤0.01, ***P≤0.001.

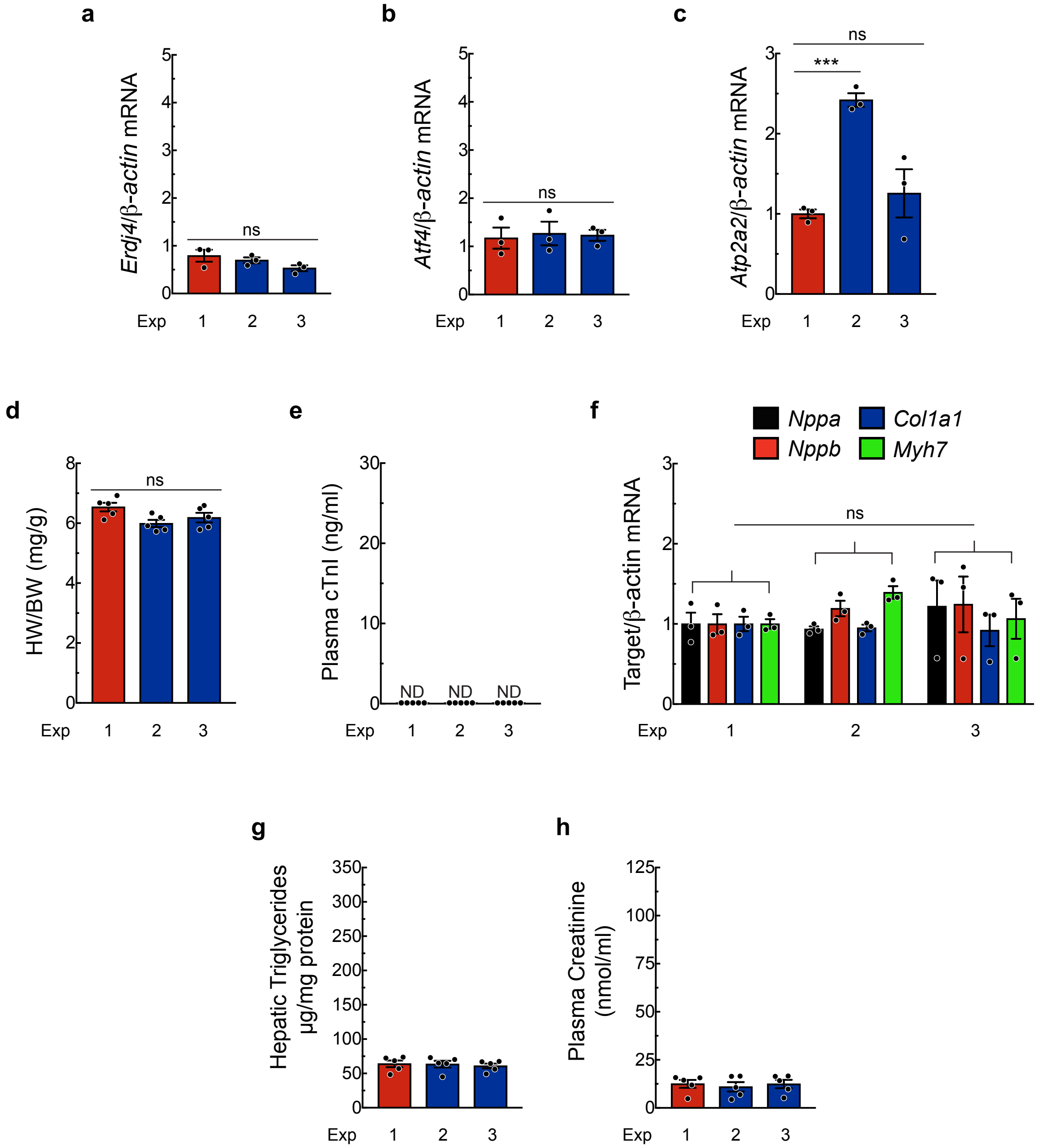

Supplementary Fig. 4 –147 exhibits no deleterious effects, in vivo.
a-c, qPCR for Erdj4 (**a**), Atf4 (**b**), and Atp2a2 (**c**) following experimental design in Fig. 6a. **d,** Ratio of heart weight to body weight (n=5). **e,** Plasma cTnI (n=5). **f,** qPCR for cardiac pathology genes: Nppa (black), Nppb (red), Col1a2 (blue), and Myh7 (green) following experimental design in Fig. 6a (n=3). **g,** Triglyceride levels in liver extracts from mice following experimental design in Fig. 6a (n=5). **h,** Plasma creatinine from mice following experimental design in Fig. 6a (n=5). Data are represented as mean ± s.e.m. Two-group comparisons were performed using Student's two-tailed t-test, and all multiple group comparisons were performed using a one-way ANOVA with a Newman-Keuls post-hoc analysis. ***P≤0.001.

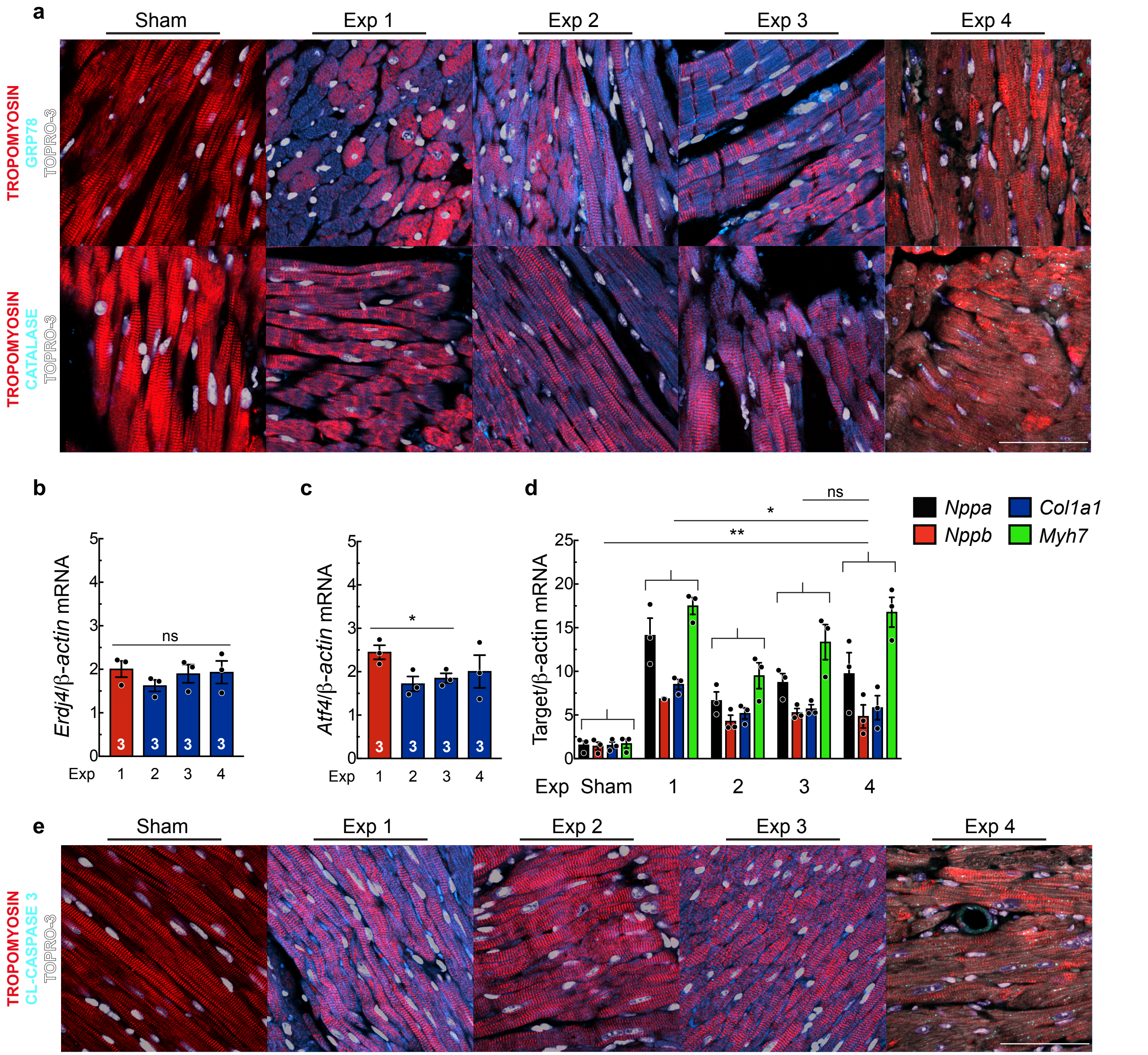

Supplementary Fig. 5 –147 decreases pathological remodeling 7d post-AMI.
a, IHC staining for GRP78 or CAT (cyan), tropomyosin (red), and nuclei (TOPRO-3) in left ventricular free wall of sham hearts or the border zone of hearts from respective trials of experimental design in Fig. 7a. Tissue sections are representative images from one mouse per condition. Scale bar represents 50μm. **b, c,** qPCR for Erdj4 (**b**) or Atf4 (**c**) in border zone of mice from Experiments 1-4 of the chronic I/R protocol shown in Fig. 7a (n=3). **d,** qPCR for cardiac pathology genes: Nppa (black), Nppb (red), Col1a2 (blue), and Myh7 (green) in border zone of mice from Experiments 1-4 of the chronic I/R protocol shown in Fig. 7a (n=3). Statistics represent significance of entire gene sets for each trial from that of separate experiments. **e,** IHC staining for cleaved caspase-3 (cyan), tropomyosin (red), and nuclei (TOPRO-3) in LV free wall of sham hearts or the border zone of hearts from indicated experiments of experimental design in Fig. 7a. Tissue sections are representative images from one mouse per condition. Scale bar represents 50μm. Data are represented as mean ± s.e.m. Two-group comparisons were performed using Student's two-tailed t-test, and all multiple group comparisons were performed using a one-way ANOVA with a Newman-Keuls post-hoc analysis. *P≤0.05, **P≤0.01.

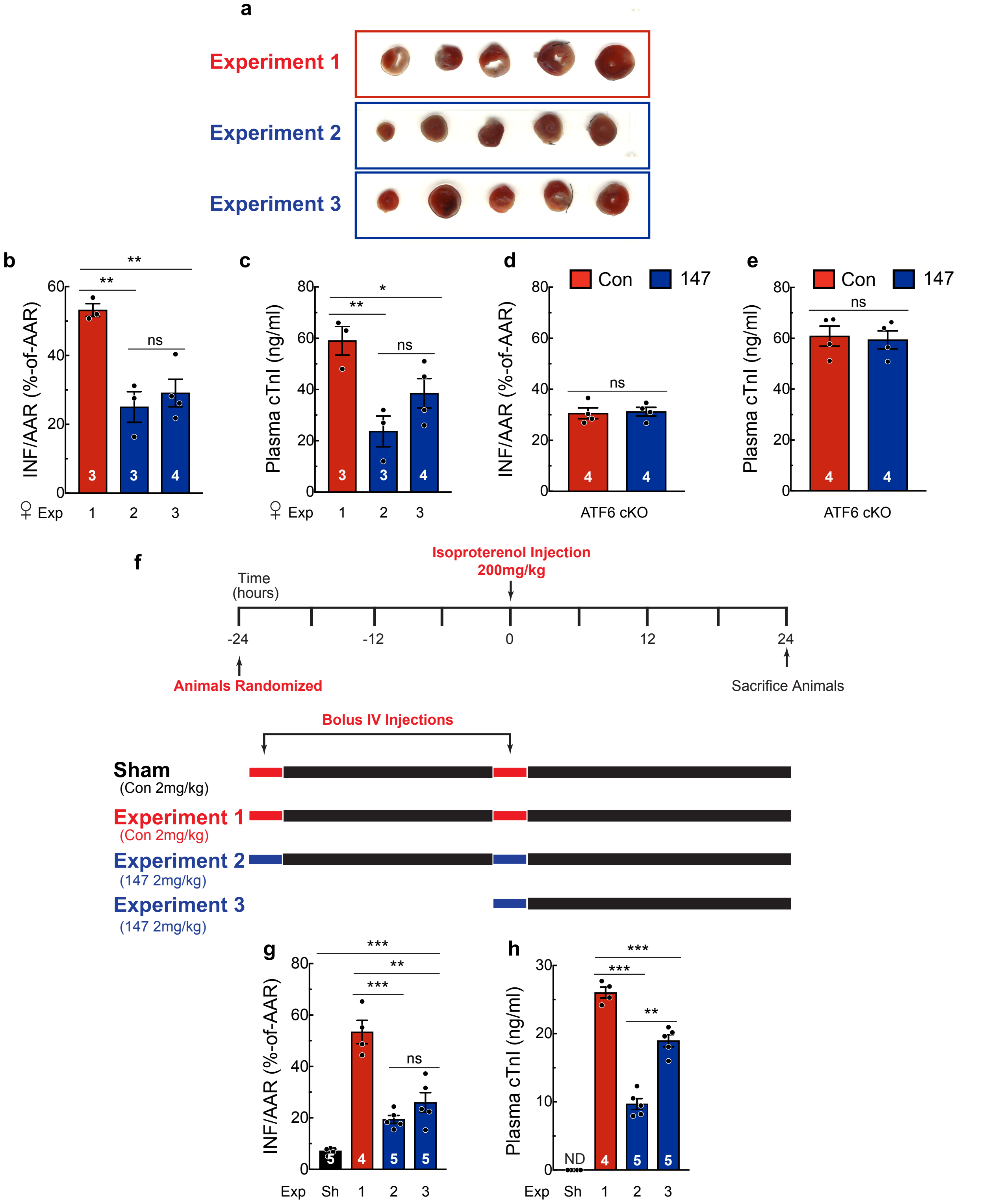

Supplementary Fig. 6 –147 is protective in multiple models of myocardial damage.

a, Representative images of TTC-stained post-I/R hearts from Experiments 1-3 of the acute I/R protocol shown in Fig. 8e. **b, c,** Relative infarct sizes (**b**) and plasma cTnI (**c**) of female mice 24-hours after reperfusion when following the acute I/R protocol shown in Fig. 6e (n=3-4 for each experiment, as shown). **d, e,** Relative infarct sizes (**d**) and plasma cTnI (**e**) of ATF6 cKO mice 24-hours post-I/R when following experimental Experiments 1 (Con) and 2 (147) of the acute I/R protocol (n=4). **f,** Experimental design for testing the effects of 147 in a different model of a AMI using isoproterenol. **g-h,** Relative infarct sizes (**g**), and plasma cTnI (**h**) (n=4-5 for each experiment, as shown). Data are represented as mean ± s.e.m. Two-group comparisons were performed using Student's two-tailed t-test, and all multiple group comparisons were performed using a one-way ANOVA with a Newman-Keuls post-hoc analysis. *P≤0.05, **P≤0.01, ***P≤0.001.

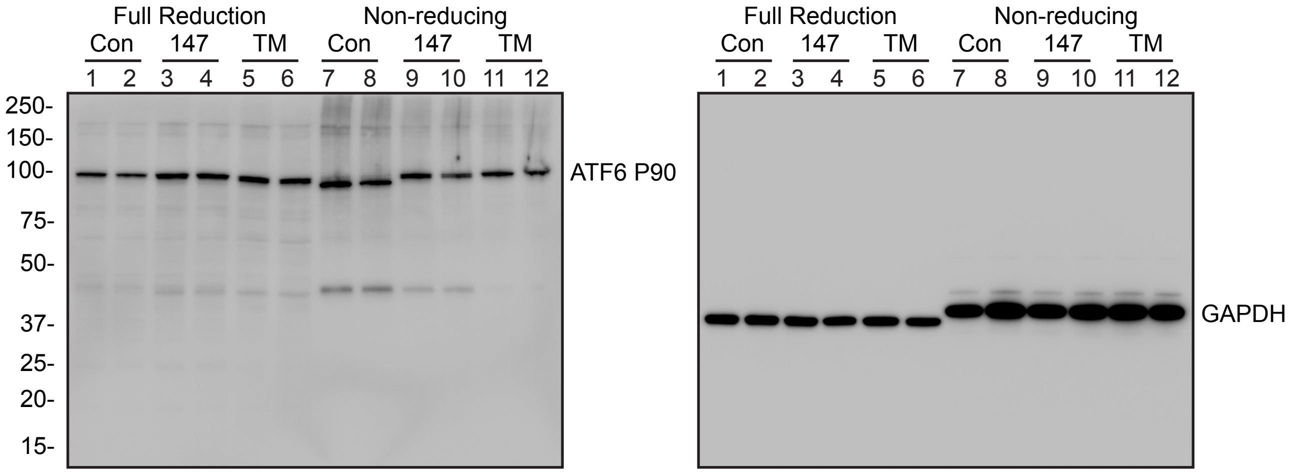

Supplementary Fig. 7 –Full uncropped gel images of Fig. 3c.

Supplementary Table I: 7-day I/R echocardiographic parameters

	WT Baseline (n = 5)	ATF6 cKO Baseline (n = 5)	WT Post-I/R (n = 5)	ATF6 cKO Post-I/R (n = 5)
FS (%)	34.17±1.74	36.09±1.55	25.43±1.38[1]	21.36±1.09[1,2]
EF (%)	64.37±2.38	67.22±1.88	51.07±2.52[1]	44.10±3.51[1,2]
LVEDV (μl)	41.46±2.83	36.03±3.95	43.69±4.34	55.36±4.78[1,2]
LVESV (μl)	14.86±1.62	11.59±1.06	17.61±4.42	32.22±3.51[1,2]
LVIDD (mm)	3.21±0.09	3.02±0.15	3.49±0.16[1]	3.77±0.27[1,2]
LVIDS (mm)	2.11±0.09	1.92±0.07	2.60±0.12[1]	2.83±0.27[1]
PWTD (mm)	1.47±0.13	1.43±0.10	0.97±0.15[1]	1.03±0.13[1]
PWTS (mm)	1.56±0.13	1.63±0.15	1.21±0.19[1]	1.17±0.14[1]
AWTD (mm)	0.90±0.05	0.92±0.08	0.72±0.04[1]	0.73±0.06[1]
AWTS (mm)	1.26±0.06	1.22±0.07	1.14±0.06	1.10±0.05[1]
LV mass (mg)	102.70±7.81	91.73±7.45	106.53±6.30	115.43±4.29[1,2]
HR (bpm)	504±9.96	540±9.99	543±7.54	546±6.24

FS = fractional shortening
EF = ejection fraction
LVEDV = left ventricular end diastolic volume
LVESV = left ventricular end systolic volume
LVIDD = left ventricular inner diameter in diastole
LVIDS = left ventricular inner diameter in systole
PWTD = left ventricular posterior wall thickness in diastole
PWTS = left ventricular posterior wall thickness in systole
AWTD = left ventricular anterior wall thickness in diastole
AWTS = left ventricular anterior wall thickness in systole
LV mass = left ventricular mass
HR = heart rate in beats per minute

Statistical analyses used a one-way ANOVA with a Newman-Keuls post-hoc analysis.

[1] = $p \leq 0.05$ different from respective Baseline
[2] = $p \leq 0.05$ different from WT Post-I/R

Supplementary Table 2: Compound 147 7-day Time Course echocardiographic parameters

	Trial 1 Baseline (n = 5)	Trial 2 Baseline (n = 5)	Trial 3 Baseline (n = 5)	Trial 1 7-day (n = 5)	Trial 2 7-day (n = 5)	Trial 3 7-day (n = 5)
FS (%)	34.00±2.56	25.34±1.58	27.68±1.90	35.21±2.17	34.44±2.11[1]	31.73±4.11
EF (%)	64.51±3.30	51.55±2.63	55.29±3.22	66.16±2.77	65.61±2.72[1]	60.51±5.83
LVEDV (μl)	30.40±6.89	33.83±6.27	32.46±5.61	30.01±2.98	21.61±1.47[1]	32.26±3.46
LVESV (μl)	10.22±1.50	16.30±3.03	14.89±3.16	10.27±1.39	7.30±0.32[1]	13.04±2.82
LVIDD (mm)	2.78±0.23	2.91±0.22	2.87±0.20	2.81±0.11	2.46±0.07[1]	2.88±0.13
LVIDS (mm)	1.82±0.11	2.17±0.16	2.08±0.18	1.82±0.11	1.61±0.03[1]	1.98±0.17
PWTD (mm)	1.66±0.08	1.40±0.19	1.37±0.14	1.17±0.11[1]	1.80±0.03[1]	1.40±0.26
PWTS (mm)	1.76±0.05	1.67±0.17	1.45±0.10	1.43±0.12[1]	2.01±0.09[1]	1.61±0.10
AWTD (mm)	1.01±0.04	1.02±0.08	0.98±0.02	0.91±0.03	0.88±0.01	0.95±0.04
AWTS (mm)	1.22±0.13	1.19±0.05	1.20±0.06	1.29±0.04	1.16±0.07	1.21±0.11
LV mass (mg)	105.21±4.51	107.38±6.23	93.15±5.72	80.24±3.78[1]	99.78±1.89	97.21±11.04
HR (bpm)	543±9.05	493±14.51	488±40.29	522±2.76[1]	515±10.32	520±5.38

FS = fractional shortening
EF = ejection fraction
LVEDV = left ventricular end diastolic volume
LVESV = left ventricular end systolic volume
LVIDD = left ventricular inner diameter in diastole
LVIDS = left ventricular inner diameter in systole
PWTD = left ventricular posterior wall thickness in diastole
PWTS = left ventricular posterior wall thickness in systole
AWTD = left ventricular anterior wall thickness in diastole
AWTS = left ventricular anterior wall thickness in systole
LV mass = left ventricular mass
HR = heart rate in beats per minute

Statistical analyses used a one-way ANOVA with a Newman-Keuls post-hoc analysis.

[1] = $p \leq 0.05$ different from respective Baseline

Supplementary Table 3: Compound 147 7-day AMI echocardiographic parameters

	Trial 4 Baseline (n = 5)	Trial 5 Baseline (n = 5)	Trial 6 Baseline (n = 5)	Trial 7 Baseline (n = 5)	Trial 4 Post-AMI (n = 5)	Trial 5 Post-AMI (n = 5)	Trial 6 Post-AMI (n = 5)	Trial 7 Post-AMI (n = 5)
FS (%)	33.08±2.45	34.91±5.58	32.22±1.39	33.58±4.77	22.60±2.39[1]	33.29±3.09[2]	28.05±1.57[1,2]	31.34±3.19[2]
EF (%)	63.42±3.64	65.05±7.21	62.21±2.18	62.24±6.29	50.40±3.75[1]	62.74±4.37[2]	57.07±4.23[2]	60.03±4.74[2]
LVEDV (μl)	25.65±2.70	31.06±4.20	31.12±4.54	46.15±3.44	45.69±2.58[1]	33.81±2.33[2]	29.44±5.22[1,2]	40.46±0.561[1,2]
LVESV (μl)	9.68±1.97	11.46±3.40	12.15±2.37	18.20±3.80	21.02±3.49[1]	15.92±4.60	10.80±7.77	15.33±2.83
LVIDD (mm)	2.63±0.11	2.84±0.16	2.83±0.17	3.35±0.11	3.43±0.15[1]	3.02±0.11[2]	2.76±0.19[2]	3.18±0.08[2]
LVIDS (mm)	1.77±0.13	1.87±0.26	1.93±0.16	2.25±0.22	2.40±0.16[1]	2.15±0.17[2]	1.85±0.12[2]	2.14±0.11[2]
PWTD (mm)	1.47±0.10	1.34±0.08	1.43±0.11	0.91±0.15	1.55±0.07	1.42±0.14	1.17±0.28[1,2]	0.99±0.15[2]
PWTS (mm)	1.73±0.08	1.60±0.19	1.65±0.11	1.35±0.16	1.69±0.04	1.89±0.22	1.63±0.23	1.32±0.16
AWTD (mm)	0.88±0.02	1.02±0.08	0.88±0.03	1.01±0.04	0.87±0.04	1.04±0.09[2]	0.83±0.08	1.16±0.15
AWTS (mm)	1.12±0.05	1.31±0.09	1.17±0.05	1.28±0.06	1.17±0.04	1.43±0.12[2]	1.15±0.06	1.45±0.20
LV mass (mg)	90.82±1.26	97.68±3.33	94.12±5.52	91.03±12.18	126.30±7.43[1]	113.39±2.43[1,2]	118.22±4.96[1]	125.57±5.74[1]
HR (bpm)	522±10.54	517±20.40	545±6.88	535±11.80	507±11.32	529±9.45	492±24.50	527±7.10

FS = fractional shortening
EF = ejection fraction
LVEDV = left ventricular end diastolic volume
LVESV = left ventricular end systolic volume
LVIDD = left ventricular inner diameter in diastole
LVIDS = left ventricular inner diameter in systole
PWTD = left ventricular posterior wall thickness in diastole
PWTS = left ventricular posterior wall thickness in systole
AWTD = left ventricular anterior wall thickness in diastole
AWTS = left ventricular anterior wall thickness in systole
LV mass = left ventricular mass
HR = heart rate in beats per minute

Statistical analyses used a one-way ANOVA with a Newman-Keuls post-hoc analysis.

[1] = $p \leq 0.05$ different from respective Baseline

[2] = $p \leq 0.05$ different from Trial 4 Post-AMI

Supplementary Table 4: Compound 147 24-hour AMI echocardiographic parameters

	Trial 8 Baseline (n = 3)	Trial 9 Baseline (n = 4)	Trial 10 Baseline (n = 4)	Trial 8 Post-AMI (n = 3)	Trial 9 Post-AMI (n = 4)	Trial 10 Post-AMI (n = 4)
FS (%)	35.07±1.61	33.01.91±2.75	30.94±2.75	34.06±2.41	34.70±1.13	30.27±1.86
EF (%)	66.14±2.43	63.07±4.04	60.61±4.18	64.60±3.49	65.61±1.35	58.99±2.88
LVEDV (μl)	32.00±8.38	30.74±3.75	23.34±2.70	29.22±3.21	32.17±3.63	39.03±5.67[1]
LVESV (μl)	11.32±3.68	11.70±2.38	9.40±1.93	10.73±2.34	10.92±0.81	16.46±3.14[1]
LVIDD (mm)	2.83±0.29	2.83±0.15	2.54±0.12	2.77±0.12	2.89±0.13	3.11±0.20[1]
LVIDS (mm)	1.85±0.23	1.91±0.16	1.76±0.15	1.84±0.14	1.88±0.05	2.18±0.19[1]
PWTD (mm)	1.40±0.16	1.38±0.25	1.60±0.08	1.30±0.13	1.27±0.04	1.31±0.07[1]
PWTS (mm)	1.61±0.13	1.78±0.17	1.77±0.10	1.66±0.13	1.61±0.08	1.53±0.09[1]
AWTD (mm)	1.07±0.03	0.95±0.07	0.97±0.12	1.10±0.03	0.97±0.04	0.97±0.11
AWTS (mm)	1.32±0.05	1.29±0.10	1.19±0.06	1.31±0.05	1.36±0.06	1.22±0.06
LV mass (mg)	129.50±7.91	120.70±17.09	122.45±6.31	125.02±5.62	119.03±11.24	136.04±4.34[1]
HR (bpm)	535±14.75	533±15.94	528±12.36	535±16.33	478±20.51	544±10.22

FS = fractional shortening
EF = ejection fraction
LVEDV = left ventricular end diastolic volume
LVESV = left ventricular end systolic volume
LVIDD = left ventricular inner diameter in diastole
LVIDS = left ventricular inner diameter in systole
PWTD = left ventricular posterior wall thickness in diastole
PWTS = left ventricular posterior wall thickness in systole
AWTD = left ventricular anterior wall thickness in diastole
AWTS = left ventricular anterior wall thickness in systole
LV mass = left ventricular mass
HR = heart rate in beats per minute

Statistical analyses used a one-way ANOVA with a Newman-Keuls post-hoc analysis.

[1] = $p \leq 0.05$ different from respective Baseline

Supplementary Table 5: qRT-PCR primers

Target	Species	Forward Sequence 5' to 3'	Reverse Sequence 5' to 3'
Grp78	Mouse	TTCTGCCATGGTTCTCACTAAA	TGTTCTTCTCTCCCTCTCTCTT
Cat	Mouse	ACCAGATACTCCAAGGCAAAG	TGGAGAATCGAACGGCAATAG
Grp94	Mouse	GGGAGGTCACCTTCAAGTCG	CTCGAGGTGCAGATGTGGG
Atf6	Mouse	GCGGATGATAAAGAACCGAGAG	ACAGACAGCTCTTCGCTTTG
Erdj4	Mouse	GGATGGTTCTAGTAGACAAAGG	CTTCGTTGAGTGACAGTCCTGC
Atf4	Mouse	CTTGCTGTCTGCCGGTTTG	GGGAAGAGGAAAGGACACCC
Atp2a2	Mouse	CCAGAGAGATGCCTGCTTAAA	CACGTTGGATGAGATGAGGTAG
Nppa	Mouse	GAGAGAGAGAAAGAAACCAGAGTG	CTCATCTTCTACCGGCATCTTC
Nppb	Mouse	GTCAGTCGTTTGGGCTGTAA	GCAAGTTTGTGCTCCAAGATAAG
Col1a1	Mouse	GAAGCACGTCTGGTTTGGA	ACTCGAACGGGAATCCATC
Myh7	Mouse	TGCCCGATGACAAAGAAGAG	AAGAGGCCCGAGTAGGTATAG
β-Actin	Mouse	GACGGCCAGGTCATCACTAT	GTACTTGCGCTCAGGAGGAG
Grp78	Human	CCACCTCAGTCTCCCAGCTAA	ACAATGGTGGTACGAGCCG
Cat	Human	GAACTGTCCCTACCGTGCTCGA	CCAGAATATTGGATGCTGTGCTCCAGG
β-Actin	Human	AAATCTGGCACCACACCTTC	GGGGTGTTGAAGGTCTCAAA
